# An Analysis of the Transfer Lengths of Different Types of Prestressed Fiber-Reinforced Polymer Reinforcement

**DOI:** 10.3390/polym14193931

**Published:** 2022-09-20

**Authors:** Aidas Jokūbaitis, Juozas Valivonis

**Affiliations:** Department of Reinforced Concrete Structures and Geotechnics, Faculty of Civil Engineering, Vilnius Gediminas Technical University, Sauletekio av. 11, LT-10223 Vilnius, Lithuania

**Keywords:** fiber-reinforced polymer, transfer length, prestress, bond

## Abstract

The main aim of this paper is to provide a broader analysis of the transfer lengths of different types of fiber-reinforced polymers (FRPs) and to provide corrections to the existing theoretical models. Therefore, this paper presents a description of the main factors that influence the transfer lengths of different types of FRPs based on experimental results found in the literature. A database of more than 300 specimens was compiled with the results of the transfer lengths of different FRPs and the main influencing parameters. The analysis of the database results showed that the transfer length of the carbon fiber composite cable (CFCC) strands depends on the type of prestressed reinforcement release. Therefore, in this article, the new coefficient α_t_ = 2.4 is proposed for the transfer length of suddenly released CFCC strands. Additionally, the transfer length of the aramid fiber reinforced polymer (AFRP) depends on its surface conditions. Therefore, new coefficients α_t_ = 1.5 and α_t_ = 4.0 are also proposed for the transfer lengths of smooth braided and sanded and rough AFRP bars, respectively. Furthermore, the proposed coefficients α_t_ = 2.6, α_t_ = 1.9, and α_t_ = 4.8 found in the literature were validated with the analysis of a larger database of the transfer lengths of glass fiber-reinforced polymer (GFRP) bars, carbon fiber-reinforced polymer (CFRP) bars, and gradually released CFCC strands, respectively. Moreover, the main existing theoretical models are presented, and the comparison of theoretical and experimental transfer length results is discussed. However, the low number of specimens prestressed with basalt fiber-reinforced polymer (BFRP) bars prevented the deeper analysis of the results. the analysis of the transfer length and the proposed new values of the coefficient α_t_ provides possibilities for adapting it to design codes for engineering applications and performing additional research that fills the missing gaps in the field.

## 1. Introduction

There has been increasing concern about the durability of prestressed concrete structures due to the corrosion of prestressed steel strands, especially in corrosive environments such as parking garages, bridges, marine structures, and railway sleepers [1,2,3]. Steel corrosion is accelerated in these structures as a result of the influence of a chloride-rich environment (deicing salts, etc.). Therefore, there is increasing interest in the use of FRP materials, namely, CFRP, AFRP, GFRP, and the relatively new BFRP [4,5] as replacements for steel reinforcements. BFRP was developed for applications in civil infrastructure as a structural reinforcement material. It has similar tensile strength, modulus of elasticity, and cost to GFRP. BFRP is more chemically stable, has a wider range of working temperatures, and is much cheaper than CFRP. Additionally, it is durable, resistant to high temperatures, corrosion, acid, radiation, ultraviolet (UV), and vibration; it also has excellent electromagnetic characteristics. Furthermore, BFRP is resistant to alkalis and is distinct from GFRP. AR–glass fibers are suggested for application in alkaline environments. However, it has a much higher cost. AFRP has good resistance to impact, high strength, high modulus of elasticity, the lowest density, and sufficient stiffness. AFRP can be utilized for impact-resistant structures. Additionally, AFRP is sufficient for application for strands and cables; however, it has low compressive strength perpendicular to the fiber direction and low long-term strength, as well as higher cost. CFRP has the highest strength of FRP materials and the largest range of strengths. CFRP is more resistant to creep rupture and fatigue failure than the other FRPs. Additionally, it has low conductivity and a high modulus of elasticity, is resistant to chemical effects, and does not absorb water as well. Its higher cost is offset by its high strength and high resistance to cyclic and fatigue failures. CFRP with an ultrahigh modulus of elasticity is popular in the aerospace industry because its strength-to-weight ratio is among the highest of all FRPs. CFRP with a normal modulus of elasticity is used in the infrastructure industry.

FRP consists of strong glass, carbon, aramid, and basalt fibers embedded in a resin matrix. Hybrid fiber reinforced polymer (HFRP) combines CFRP and GFRP [6,7]. The quality of the composite material depends on the surface roughness and the adhesion between the filler and the matrices, which can be enhanced with additional treatment [8,9]. FRP is an anisotropic material (contrary to steel), resulting in better mechanical properties in the longitudinal direction (Table 1). FRP has a lighter weight and higher strength. However, its mechanical properties are linear elastic, which leads to a lower failure strain and elongation rate. Additionally, the modulus of elasticity of FRP is lower than that of steel (except for CFRP bars with a high modulus of elasticity). The material properties of FRPs depend on the fibers, matrices, and fiber–volume ratios. FRP is resistant to corrosion, which can significantly increase the service life of the structure.

FRPs have additional important properties that make them particularly attractive for prestressed concrete applications: high strength, which is similar to or greater than that of steel and low modulus of elasticity, which results in lower concrete prestress losses due to concrete creep and shrinkage as well as the relaxation of the prestressing element.

The major difficulty in using FRP reinforcement for prestressing is that anchorage systems require greater attention than those for steel strands. Therefore, three types of anchorage systems are developed for FRP reinforcement: mechanical, bonded, and composite [9].

The mechanical anchorage of reinforcement consisting of a steel barrel and wedges is ensured by friction between the reinforcement surface and the inner conical anchorage surface. Therefore, compressive stresses appear perpendicular to the reinforcement. However, FRP is an anisotropic material that has weak material properties perpendicular to the fibers. Therefore, practitioners suggest enhancing the mechanical anchorage of the FRP reinforcement using a small slope angle between the inner surface of the barrel and the outer surface of the wedges, the rounded edges of the wedges, and a soft metal sleeve (aluminum or copper) around the FRP. Other mechanical anchorages were described in [9,15] such as the curved wedge anchorage system [16], the nonmetallic wedge anchorage system [17], the integrated sleeve-wedge anchorage system [18], the spike anchorage system, and the clamping anchorage system. The mechanical anchors are usually of small size. However, this type of anchorage lacks stability and reliability.

The bonded-type anchorage consists of a steel barrel with a bonding material (cementitious or resin) surrounding the FRP bar. The inner part of the barrel can have three different shapes: straight; conical with decreased length but larger diameter; or straight and conical with increased length and similar diameter with the purpose of relieving the stress concentration. The bonded-type anchorage depends on the bonding force between the CFRP cable and the bonding material to balance the tension of the cable. The performance of the bonded-type anchorage is mainly influenced by the dimensions and configuration of the steel barrel, the material type and surface condition of the FRP, and the properties of the bonding material. The main drawbacks are a large size, long curing time, creep of the bonding material, sensitivity to moisture, and high temperature.

The composite anchorage takes advantage of mechanical and bonded-type anchorage. In [19], the straight steel pipe filled with resin was directly clamped with a split-wedge anchor that improved the anchorage of the FRP. Additionally, [20] proposed a compound-type anchorage system that adapted load transfer through wedge extrusion and epoxy bonding. The composite anchorage is smaller than the bonded anchorage but larger than the mechanical anchorage and presents good anchoring performance. At the same time, it has complicated construction and installation processes. Mechanical and bond anchorage systems are simpler than the composite anchorage system. Therefore, the mechanical and bond anchorage systems are still under investigation.

The use of FRP reinforcement in prestressed concrete structures is highly dependent on the reliability of the reinforcement anchorage zone. That is, the behavior of concrete members is mainly dependent on the bond between reinforcement and concrete [21,22,23]. In pretensioned concrete structures, the prestressing force is transferred to the concrete by the bond between reinforcement and concrete, which depends on three mechanisms: adhesion, Hoyer effect (wedge action), and mechanical interlocking. The bond of prestressed FRP is mainly influenced by the large Poisson ratio related to the Hoyer effect and the high axial strain capacity of the FRP materials [24]. Furthermore, if the effect of Poisson’s ratio is dominant, the surface conditions of the FRP will have little influence on the bond [25]. In the case of FRP materials, the chemical bond (adhesion) is particularly weak [24]. Lastly, mechanical interlock and friction are quite dependent on the surface characteristics of the reinforcement.

In pretensioned members, the length from the end of the member where the tendon stress is zero to the point along the tendon where the prestress is fully effective is called the transfer length [12]. Ref. [11] defined the transfer length as the length required to transfer the prestressing force to the concrete after the release of a prestressing reinforcement. In other words, it is the distance along the member in which the effective prestressing force is developed.

One of the most important factors that affect the transfer length of prestressed reinforcement is the Hoyer effect, which is caused by the swelling of the strand in the transfer zone after release as a result of the Poisson ratio. During the transfer, the induced confining stresses normal to the tendon enhance the bond strength at the interface, as the lateral deformation is resisted by the surrounding concrete. Enhancement of the bond caused by the Hoyer effect is directly related to the strength of the concrete and the coefficient of friction between the two materials. Additionally, according to [26], the distribution of the bond stress over the transfer length is not linear due to the changing values of transverse strain (Hoyer effect) and slip over the transmission length.

Knowledge of the transfer length is essential to maintaining the integrity of the structure and to preventing the bond slip failure of the member. Additionally, an accurate estimate of the transfer length is important for checking the stress limit at the serviceability limit state, determining the actual bond stress in the anchorage zone of the pretensioned reinforcement, and designing the shear of the prestressed members. The underestimation of the transfer length leads to unconservative shear calculations, while overestimation leads to unconservative stress calculations at the serviceability limit state.

The simulation of the anchorage zone of FRP reinforcement introducing the finite element method (FEM) allows for performing a deeper analysis of the transfer length results that are difficult to measure in real experimental research. The simulation of the transfer lengths of BFRP [27], CFCC, and AFRP [28] showed that the optimization of the transfer length prediction of the FRP can be performed, eliminating unnecessary and expensive future experimental research.

In the last 30 years, numerous experimental researchers have investigated the transfer lengths of prestressed FRP. The transfer length is a function of several factors that depend on the properties of both the prestressing reinforcement and the concrete. Therefore, many studies were performed to investigate the influence of concrete compressive strength [29,30,31,32,33,34,35,36,37,38,39], prestress level [29,30,31,32,33,36,37,39,40,41,42,43,44,45,46,47], reinforcement diameter [24,26,29,30,31,32,35,36,37,38,48], concrete cover [26,29,30,31,32,35,48,49,50], reinforcement surface conditions [24,35,36,37,38,47,48,50], and modulus of elasticity [33,36,47,51,52,53,54] etc. on the transfer length of prestressed FRP reinforcement. However, the large variation in FRP bars in terms of shape, surface conditions, strength, and modulus of elasticity indicates that a deeper understanding of the transfer length of FRPs with different properties remains necessary. Therefore, this article presents a database of transfer length results for pretensioned FRP and provides a comparative analysis of the anchorage zones of different types of FRP.

## 2. Parameters That Influence the Transfer Length

The transfer length is an important parameter for the anchorage zone of prestressed reinforcement. In addition, it depends on various mechanical and geometric properties of the material. Therefore, this section presents an overview of the influence of different parameters on the transfer length of different prestressed FRP reinforcements (GFRP, CFRP, AFRP, CFCC and BFRP). Unfortunately, only a few studies on the transfer length of BFRP bars were found in the literature [27,55,56]. The lack of results and similar experimental parameters do not allow for qualitatively determining the influence of different parameters on the transfer length of BFRP bars.

### 2.1. Effect of Concrete Compressive Strength

Many authors investigated the influence of concrete compressive strength at the time of reinforcement release on the transfer length of concrete specimens pretensioned with different types of FRP reinforcement. Table 2 provides a summary of the average transfer length results, showing the influence of concrete compressive strength. The lower (f_ci.min_) and higher (f_ci.max_) concrete strength correspond to higher (L_t.max_) and lower (L_t.min_) transfer length, respectively.

Zou [33] concluded that compared with the effect of the level of prestressing, the concrete strength at transfer is a more significant factor that affects the transfer length of the spirally indented CFRP Leadline bar. Additionally, [33] stated that all tested beams prestressed with AFRP bars had similar transfer lengths but different concrete strains with respect to concrete strength. Therefore, it was concluded that the transfer length of the AFRP bars is not significantly affected by the concrete strength.

The authors of [36,37] concluded that the transfer length of the prestressed GFRP bars decreased by about 32% when the concrete strength at the time of reinforcement release increased by 2.3 times. Additionally, Crossett et al. [55] showed that the transfer length of the BFRP bar decreased by 10% with an increase in concrete strength by 43%.

The FEM model of Motwani and Laskar [28] showed that peak elastic strain of concrete at the level of prestressed CFCC and AFRP Arapree reinforcement decreases with an increase in concrete strength.

Based on [33,38,39], it was stated in [36,37] that the concrete strength had a minimum effect on the transfer length of the AFRP reinforcement due to its small diameter (normally ≤9.5 mm) and shorter transfer length. Additionally, the authors of [36,37] stated that the larger diameter of the GFRP bars (Ø12–Ø16 mm), the longer the transfer length, allowing the effect of concrete strength on the transfer length to be apparent. However, in [38,39], a higher diameter of AFRP bars was also used (Ø12–Ø16 mm), and it resulted in transfer lengths almost twice as long as in [36,37] for GFRP bars with similar prestress levels and concrete strengths. Although the modulus of elasticity of AFRP and GFRP bars is similar, the surface of AFRP bars is braided (almost plain), and it is ribbed for GFRP bars. Therefore, the different surface conditions of AFRP and GFRP reinforcement may be the deciding factor for the difference in transfer length.

According to Table 2, it can be stated that with the increase i concrete compressive strength, the FRP transfer length decreases. However, the rate of decrease of the transfer length is lower for AFRP and GFRP reinforcement compared with that for CFCC and CFRP reinforcement. Therefore, the concrete strength is more significant for CFCC and CFRP reinforcement.

### 2.2. Effect of Prestress Level

The FRP reinforcements for prestressed concrete members can have different tensile strengths and initial stresses. Therefore, it is better to compare the ratios between initial stresses and tensile strength (f_pi_/f_pu_) of different types of FRPs with the transfer length instead of the initial stresses (f_pi_). Table 3 provides a summary of average transfer length results showing the influence of prestress level on the FRP reinforcement. The lower (f_pi_/f_pu_ (min)) and higher (f_pi_/f_pu_ (max)) prestress levels correspond to the lower (L_t.min_) and higher (L_t.max_) transfer lengths, respectively.

Research by other authors revealed that the influence of prestress level gives contradictory results depending on the type of FRP. For CFRP Leadline bars [33,40,41,42] and CFRP sanded bars [43,44], the transfer length increases with the increase in prestress level (Table 3). However, other authors [31,32,39,45,46] determined that the transfer length of CFRP sanded bar [45] and the CFRP Leadline bar [31,32,39,46] is not significantly influenced by the prestress level (Table 3).

For the Ø12.5 mm CFCC strand and the concrete strength of 28–30 MPa, the average transfer length increased by 26% with an increase in the prestress level of 25%. For the Ø15.2 mm CFCC strand and the concrete strength of 22–24 MPa, the transfer length increased by 25% with an increase in prestress level of 19%. Therefore, the authors [31,32] stated that the prestress level can have a greater influence on the transfer length of the CFCC strand than the concrete compressive strength due to similar concrete strengths and a greater reduction of the transfer length at a higher prestress level. However, the results from [29,30,39] showed that the transfer length of CFCC strands is not significantly influenced by the prestress level.

Ehsani et al. [47] calculated that the transfer length of the AFRP Arapree bars (Ø9.9 mm) with a smooth surface increased by 81% with an increase in the prestress level of 43% to 59% with concrete strength of 29–31 MPa.

The authors of [36,37] tested concrete beams prestressed with GFRP bars with ribbed surfaces and calculated that for Ø16 mm GFRP bar and concrete strength of 29–31 MPa, the increase in prestress level from 26% to 41% induced a 32% increase in transfer length.

It is evident that in most cases for CFRP Leadline bars, CFCC strands, AFRP, and GFRP bars, the transfer length is proportional to the prestress level (Table 3).

### 2.3. Effect of Reinforcement Diameter

Several authors have investigated the influence of reinforcement diameter on the transfer length of pretensioned FRP reinforcement. Table 4 provides a summary of average transfer length results showing the influence of reinforcement diameter. The lower (Ø_min_) and higher (Ø_max_) reinforcement diameters correspond to the lower (L_t.min_) and higher (L_t.max_) transfer lengths, respectively.

Domenico and colleagues [29,30] and Mahmoud and colleagues [31,32] calculated that the transfer length of CFCC strands increased by 9–34% and 32%, respectively, with an increase in reinforcement diameter from 12.5 mm to 15.2 mm. Stark and Hegger [26] tested UHPFRC (ultra-high-performance fiber-reinforced concrete) beams prestressed with CFCC 7-wire helical indented strands (Ø7.5 mm) and CFRP spirally indented bars (Ø5 mm). The test results showed that the transfer length of the CFCC strand was 73% higher than the CFRP bar. As both reinforcements had indented surfaces, the helical form of the CFCC 7-wire strand may have an additional positive influence on bond conditions.

Nanni and colleagues [24,38] calculated that at a low prestress level (24%), the transfer length increased by 38% with an increase in the diameter of the AFRP bar (with a smooth braided surface) from 8 mm to 12 mm. For a higher prestress level (about 50%), the transfer length increased to 42% with an increasing diameter of the AFRP bar from 8 mm to 16 mm. Furthermore, Taerwe and Pallemans [35,48] showed that the transfer length increased by 53% with an increasing diameter of the AFRP Arapree bar (with sanded surface) from 5.3 mm to 7.5 mm (the cross-sectional area of reinforcement increased twice).

In [36,37], it was calculated that with the increase in GFRP bar diameter from 12 mm to 16 mm, the average transfer length increased by 19%.

The FEM model of Motwani and Laskar [28] showed that the peak elastic strain of concrete at the level of prestressed CFCC and AFRP Arapree reinforcement increases with an increase in reinforcement diameter.

The results for the influence of reinforcement diameter on the transfer length of pretensioned CFRP, CFCC, AFRP, and GFRP reinforcement showed that it is generally consistent with the literature and that the transfer length is directly proportional to the diameter of the prestressed FRP reinforcement. However, expressing the transfer length in terms of the bar diameter only can result in inaccurate results, as it also depends on the prestressing level and the concrete compressive strength.

### 2.4. Effect of Concrete Cover

The effect of concrete cover is important for the durability of prestressed concrete structures. However, the FRP reinforcement covers this aspect. Therefore, a sufficient concrete cover is more relevant to controlling concrete splitting during the release of pretensioned reinforcement. Additionally, it is more relevant to analyze the influence of concrete cover on the cracking of the anchorage zone and the transfer length of the FRP reinforcement through the ratio between the concrete cover and the reinforcement diameter (c/Ø).

In [29,30], it was concluded that concrete cover c/Ø of 4 and 5 had no significant influence on the transfer length results for the CFCC strand sizes of Ø12.5 mm and Ø15.2 mm, respectively, for concrete strength of 30–56 MPa and prestress level of 50–75% for a given range of concrete cover (from 50 mm to 75 mm). According to [31,32], a concrete cover of 4·Ø is sufficient to prevent concrete splitting of the anchorage zone of the CFCC strand and the CFRP Leadline bar during reinforcement release for concrete strength between 22 MPa and 42 MPa, reinforcement diameter of 8–15.2 mm, and prestressing level of 60–80%. Therefore, with sufficient concrete confinement, all prestress force can be transferred to the concrete. However, all tested beams had a debonded length of 50 or 100 mm at the end of the beam. Therefore, the radial pressure moved deeper into the beam and could increase the splitting resistance of the transfer zone. Additionally, Stark and Hegger [26,49] calculated that a concrete cover c/Ø of at least 3.0 (absolute concrete cover 22.5 mm) is required for CFCC strand (Ø7.5 mm) and a CFRP bar (Ø5 mm) c/Ø of at least 4.0 (absolute concrete cover 20 mm) is required to ensure the crack-free introduction of the prestressing force for UHPFRC concrete beams.

Taerwe and Pallemans [35] calculated that for sand-coated AFRP bars (Ø5.3 mm and Ø7.5 mm), the critical concrete cover to be used to avoid concrete splitting is approximately equal to 2.8 times the bar diameter (2.8·Ø) for concrete strength of 54 MPa and prestress level of 50%. Additionally, the test results showed that the transfer length decreased with an increase in c/Ø up to approximately 3.5. The further increase in concrete cover did not have a significant influence on the transfer length of the AFRP bars.

Khin et al. [50] performed tested the minimum concrete cover (5 mm, 10 mm, and 15 mm) of concrete prisms (100 × 100 × 600 mm) pretensioned with CFRP, AFRP (prestress level of 60%), and GFRP (prestress level of 50%) bars (Ø8 mm). Based on the experimental test results, the recommended minimum concrete cover was 15 mm (c/Ø = 1.9) for the CFRP bar, but for the GFRP and AFRP bars, the concrete cover could be greater than 15 mm (c/Ø > 1.9). This could be related to the lower modulus of elasticity of the GFRP and AFRP reinforcement, which induces higher splitting stresses in the anchorage zone due to the Hoyer effect.

Motwani and Laskar [28] performed simulation tests for the anchorage zone of the prestressed CFCC strand and AFRP Arapree bar. They established that significant amounts of plastic strain appeared near the prestressing reinforcement, indicating concrete cracking at transfer for Ø12.7 mm, c = 30 mm, and c/Ø = 2.4. Additionally, they determined that the peak elastic strain of concrete at the level of prestressed CFCC and AFRP Arapree reinforcement decreases with an increase in concrete cover.

The small concrete cover could lead to the splitting of the concrete at transfer due to the insufficient confinement of the concrete. Therefore, the additional confinement of the concrete can reduce the risk of splitting the concrete during reinforcement release.

### 2.5. Effect of Shear Reinforcement

As stated in the previous section, additional concrete confinement can reduce the risk of concrete splitting during the release of pretensioned FRP reinforcement and therefore have a positive effect on the transfer length. Several authors have investigated the different types of concrete confinement at the anchorage zone of pretensioned FRP reinforcement. Table 5 provides a summary of the average transfer length results showing the influence of shear reinforcement.

Saeed [45] tested concrete beams prestressed with Ø12.7 mm CFRP sand-coated and helically wrapped bars. The distance between shear reinforcement of 75 mm and 50 mm for the level of prestressing of 50% and 55% for beams B2-4-55 and B3-4-60, respectively, was analyzed. The shorter distance between the shear reinforcements increased the confinement and decreased the transfer length (495 mm) in beam B3-4-60 compared with the transfer length (533 mm) in beam B2-4-55 despite the higher prestress level in beam B3-4-60. Finally, it was concluded that confinement, represented by the number of stirrups, played a significant role in the bond behavior between the concrete and CFRP bars [45]. Mahmoud and colleagues [31,32] calculated that for the Ø12.5 mm CFCC strands, the addition of shear reinforcement reduced the average transfer length by 21%. For the Ø8 mm CFRP Leadline bar, the addition of shear reinforcement reduced the transfer length by 6%.

Nanni et al. [57] investigated the influence of different levels of concrete confinement on the splitting of concrete during the release of smooth braided AFRP bars. It was calculated that cracking of the anchorage zone can be avoided by using a small-pitch (25 mm) CFRP coil (length 225 mm) that confines the concrete immediately surrounding the AFRP bar or partially blanketing the AFRP bar, intermittently applying the tape for lengths of one and two tendon diameters (16 and 32 mm) at a spacing of one tendon diameter over total lengths of 240 and 272 mm, respectively. However, shear reinforcement (Ø6 mm) (distance between stirrups 20 mm and 25 mm) does not prevent cracking. The transfer lengths of the specimens with shear reinforcement, CFRP coil, and partial blanketing were 625, 600, and 700 mm, respectively. Partial blanketing was determined to be very effective due to the reduction in tensile stress field despite an increase in transfer length of up to 17%.

Despite the low difference between prestress level and concrete compressive strength, it can be stated that specimens with shear reinforcement can induce a reduction in the transfer length of CFCC strands and the CFRP Leadline bar [31,32] (Table 5). Additionally, it was concluded in [31,32] that the absence of shear reinforcement in specimens pretensioned with CFRP Leadline and CFCC strand increases the transfer length with an averages of 10% and 17%, respectively, more than the values predicted by the proposed theoretical models.

### 2.6. Effect of Reinforcement Surface Conditions

The bond properties between the reinforcement and concrete are influenced by different surface roughnesses of the reinforcement. Furthermore, reinforcement surface conditions can enhance the anchorage zone behavior of pretensioned reinforcement. Table 6 provides a summary of average transfer length results that shows the influence of reinforcement surface conditions.

Ehsani et al. [47] investigated three different types of AFRP bars. The lowest transfer length (327 mm) was calculated for the AFRP Technora bar with a rough (ribbed) surface. For the AFRP Fibra bar with a smooth braided surface, the transfer length was longer (339 mm), and the transfer length of the AFRP Arapree bar with a smooth (plain) surface was the longest (385 mm). Despite the differences in reinforcement diameter (Ø7.4–10.4 mm), it can be concluded that with increasing surface roughness of AFRP reinforcement, the transfer length decreases.

Additionally, in [35,48], three different types of surface finishing (sanded, “expancel” and expancel sanded) for a Ø5.3 mm AFRP Arapree bar were investigated. The expancel coating was applied as a thin compressible layer around the bar to absorb part of the radial expansion of the bar due to thermal expansion and the Hoyer effect [35,48]. The test results revealed that the transfer length of the AFRP bars with the expancel coating (smooth surface) was greater by about 60–80% compared with the transfer length of the AFRP bars with the sanded and expancel-sanded surfaces. Additionally, the reduction of the consequences of the Hoyer effect by applying an expancel coating to the AFRP bar reduces the c/Ø up to approximately 2.33·Ø [35,48]. This indicates that the sand coating of the AFRP bars can significantly decrease the transfer length. However, it can increase the risk of concrete splitting during reinforcement release; additional concrete confinement can reduce the risk of splitting concrete.

Nanni and colleagues [24,38] calculated that the transfer length (225 mm) of sand-coated AFRP bars (Ø12 mm) was more than half the transfer length (450 mm) of smooth braided AFRP bars (Ø12 mm) when the prestress level was 48%. Although the concrete strength of sand-coated AFRP bars (39 MPa) was higher compared with the braided AFRP bars (34 MPa), the silica sand on the AFRP bars with respect to that of smooth bar increased the bond characteristics and significantly reduced the transfer length of the AFRP bar.

The test results revealed that the ribbed surface of the GFRP bar [36,37] and the sand-coated surface of the AFRP bar [24,38] give similar transfer length results (221 mm and 225 mm, respectively) despite different material properties (modulus of elasticity of 60 GPa and 68 GPa, concrete strength of 31 MPa and 39 MPa, and prestress level of 36% and 48%, respectively).

The test results of Khin et al. [50] showed that CFRP reinforcement with increased surface roughness (spirally indented, sanded, and cross-wounded) had a shorter transfer length (300 mm) than CFRP reinforcement with a helical plain surface (400 mm). Additionally, AFRP and GFRP spirally indented bars had similar moduli of elasticity (54 GPa and 47 GPa respectively) and the same transfer length (500 mm).

### 2.7. Effect of Modulus of Elasticity of Reinforcement

The difference in the bond characteristics in the anchorage zone of reinforcement can be attributed to the different moduli of elasticity of different FRP reinforcements. A lower modulus of elasticity causes more longitudinal deformation during prestressing and consequently more transverse deformation due to the Poisson ratio during the release of reinforcement for the same prestress level. Higher transverse deformations of FRP reinforcement with a lower modulus of elasticity improve bond strength at the transfer zone due to the lateral expansion of the bar, creating a wedge action known as the Hoyer effect. Table 7 provides a summary of the average transfer length results showing the influence of modulus of elasticity of reinforcement.

According to [51,52], the bond characteristic of the GFRP reinforcement is superior to that of steel. This can be attributed to the lower modulus of elasticity of the GFRP reinforcement. It could also be due to the better adhesion and interlock between the GFRP reinforcement and the concrete. Thus, for the same force, the transfer length for the GFRP reinforcement is smaller. Additionally, Zawam and Soudki [36] stated that the lower modulus of elasticity of the GFRP bar will induce more radial expansion compared with CFRP, resulting in an increase in the confining stresses normal to the tendon during the prestress transfer process. This characteristic improved the bond strength at the interface between the bar and the surrounding concrete upon release, resulting in a shorter transfer length compared with CFRP bars [36].

Zou [33] calculated that the transfer length of CFRP Leadline bars (Ø8 mm) was 2–3.8 times higher compared with the transfer length of the AFRP Arapree sanded bar (Ø7.8 mm). The large difference in transfer length can be attributed to the significantly lower modulus of elasticity of the AFRP bar (54 GPa) compared with the CFRP Leadline bar (172 GPa). Additionally, the sanded surface of the AFRP bar can enhance bonding conditions, in contrast with the spirally indented CFRP Leadline bar.

Ehsani et al. [47] calculated that the transfer length of the CFCC (helical plain surface) and CFRP Leadline (spirally indented surface) reinforcement was similar (432 mm). However, the transfer length of the AFRP Technora bar (rough surface) was shorter (327 mm) and may be related to the higher surface roughness and lower modulus of elasticity (69 GPa) of the AFRP bar compared to the CFCC strand and CFRP bar.

Lu et al. [53,54] calculated that the transfer length of the AFRP Technora bar (rough surface) was 13% shorter compared with the CFRP Leadline bar (spirally indented surface). It can be related to a higher surface roughness and a lower modulus of elasticity (45 GPa) of the AFRP bar compared to the CFRP Leadline bar (171 GPa).

Since the modulus of elasticity of FRPs is generally less than that of steel, the transfer length of most FRPs is less than that of steel [11].

### 2.8. Dead End versus Live End for Transfer Length Results

Different transfer lengths may appear at two ends of the pretensioned concrete member. This can be attributed to more than one specimen prestressed with the same reinforcement. In this case, one end of the reinforcement is prestressed (Live end), while the other at the same time is fixed to the rigid support (Dead end). During the release of prestressed reinforcement, differences can appear in the transfer lengths of different FRP reinforcements.

In [58], the measured transfer lengths at the live and dead ends of the box beams were in close agreement with the sudden release of the CFCC strands and CFRP Leadline bars. The same trend was identified for the gradual release of CFRP Leadline bars [45], AFRP bars [24], and GFRP ribbed bars [36,37].

The experimental results provided in [59,60,61] showed that the transfer lengths of the CFCC strands at the live ends are higher than the transfer lengths at the dead ends for the sudden release. As explained by [62], this could be influenced by factors such as concrete casting location, cutting location, and the use of multiple batches of concrete. However, the average dead end to live end ratios of transfer lengths ranged from 0.74 for pile 3 to 0.86 for piles 4 and 5. This shows that the casting location had minimal to no effect on the transfer length. Additionally, Krem [43] calculated that the transfer length at the live end was slightly greater than at the dead end for the same beam. The increase in transfer length at the live end could be a result of the dynamic impact of the sudden release of the CFRP bar.

The results of Crossett et al. [55] showed that the transfer length of the BFRP bars is 16–48% higher at the live end than at the dead end. Additionally, it was explained that the release of stress is more gradual at the dead (anchoraged) end, and therefore, shorter transfer lengths were calculated [53].

### 2.9. Long-Term Effect

The transfer of the prestressing force to the concrete during the release of reinforcement is a short-term effect. However, the prestressed concrete member is intended for long-term operation. After the release of the prestressed reinforcement, the concrete members are affected by many long-term effects (shrinkage and creep of the concrete, relaxation of the reinforcement, etc.) that can influence the anchorage zone and transfer length of the prestressed concrete member.

Zou [33] stated that the transfer length of AFRP Arapree sanded bars is not affected by time (up to 238 days), although the average concrete strain beyond the transfer length increases significantly. Nanni et al. [24] have also drawn the same conclusion for smooth braided AFRP bars for up to three weeks.

According to Zou [33], there is no apparent deterioration of the bond between the CFRP Leadline bar and the concrete with time, and the measured transfer length did not change with time for normal and high strength concrete beams. Furthermore, the application of two-point loads in the midspan did not affect the transfer length up to 390 days.

Grace [39] showed that the increase in the transfer length of CFRP Leadline bar after 300 days and the CFCC strands after 391 days is only about 7.8% and 7%, respectively, and there is no significant effect of time on the transfer length. Approximately the same result (6%) is for steel strands [63].

Soudki et al. [46] calculated that 200 days after CFRP Leadline bar (prestress level of 50%) release for the T-beam specimen, no significant change in the measured transfer length was observed.

Lu et al. [54] also determined that there are little changes in transfer length with time for CFRP Leadline and CFRP (both spirally indented) and bars after 28 days and for AFRP Teachnora (rough) bars after 90 days.

Mahmoud et al. [31,32] determined that the helical shape of the CFCC and steel strands enhanced the mechanical component of the bond and did not result in an increase in the transfer length over time. The 22% increase in the transfer length of CFRP Leadline bar over time could be due to its relatively smooth surface compared to CFCC and steel strands.

In [51,52], it was calculated that the transfer length increased by 40% and 20% with time (after 600 days) for the GFRP and steel strands, respectively. However, the rate of increase was almost double for GFRP reinforcement compared with steel.

According to [55], the transfer length of the BFRP bars increased after 5 days of reinforcement release by 4–39%.

It is suggested that at the ends of the member, the concrete strain increased with an increase in time, mainly due to the shrinkage of the concrete [46]. Away from the ends, the increase in strain includes the combined effects of creep, shrinkage, and the relaxation of the prestressing reinforcement [24,33,46].

## 3. Theoretical Models

Table 8 presents a summary of the recommended expressions for the transfer length of the FRP reinforcement (Equation (1)–(3)) and steel strands (Equations (4)–(6)). The nomenclature in this table is presented.

Equation (5) provided in [64] is evaluating the fewest different parameters (f_pi_ and Ø) influencing the transfer length. Additionally, with an empirical coefficient (20.7) which is based on the large database of transfer length results, Equation (5) was developed for steel strands. Mitchell et al. [63] suggested supplementing the ACI-318 [64] equation with the compressive strength at transfer. As concrete strength enhances the bond of reinforcement, it becomes a good additional parameter for increasing the accuracy of the transfer length prediction. Additionally, Equation (6) with the empirical coefficient (20.7) is proposed for the steel strands. Equation (3) proposed by Domenico [30] replaces the reinforcement diameter (Ø) with a cross-sectional area (A_p_) of the reinforcement and proposes an empirical coefficient C_T_ = 80 for the CFCC strand. Additionally, it calculates $f_{ci}^{1/2}$ as also in Equations (2) and (6). However, Zou [33] stated that the influence of prestress on the transfer length of CFRP reinforcement is significantly lower than the concrete strength at transfer. Therefore, it can be neglected, and the transfer length can be calculated according to Equation (2) with an empirical coefficient κ = 480 (in N·mm units) for the prestressed CFRP Leadline bar. According to [58,65], the theoretical results of the transfer length of the CFRP bar predicted by Equations (1) and (5) were up to 10% and 6% higher compared with the experimental ones, respectively. For CFCC strands [58,66], the theoretical values predicted by Equations (1) and (5) were up to 15% higher and up to 12% lower compared with the experimental results, respectively. Furthermore, Equation (2) provided the least inaccurate prediction and the theoretical values of the transfer length of the CFRP and CFCC reinforcement were up to 2.8 and 2.9 times higher than the experimental ones, respectively. Therefore, these results contradict the concluding remarks of Zou [33], who states that for all practical purposes, the effect of prestress on the transfer length of the tendons can be neglected. Based on the measured data in [31,32], the transfer length of CFRP reinforcement is proportional to the reinforcement diameter, the initial prestress level, and the concrete compressive strength at transfer, and Equation (1) was proposed for the transfer length of CFRP reinforcement. Equation (1) was adopted in design codes [10,12]. The main difference from other theoretical models is that Equation (1) proposes an empirical coefficient α_t_ dependent on the type of FRP reinforcement. Therefore, the coefficient α_t_ can be calibrated for different types of FRP reinforcement (GFRP, CFCC, CFRP, AFRP, BFRP) with different surface conditions. Additionally, it presents the concrete strength as $f_{ci}^{2/3}$ instead of $f_{ci}^{1/2}$ (Equations (2), (3) and (6)). The presentation of concrete strength as $f_{ci}^{2/3}$ can be explained by the correlation of concrete compressive strength with concrete tensile strength $f_{ctm}=0.3\cdot f_{ci}^{2/3}$ provided in [67,68,69]. Additionally, Marti-Vargas et al. [70] proposed Equation (4) for prestressed seven-wire steel strands, which is similar to Equation (1). The only differences are in the empirical coefficient (2.5) and that the reinforcement diameter (Ø) is replaced with an area of cross-section of reinforcement (A_p_).

Table 8 presents a summary of the recommended expressions for the transfer length of the FRP reinforcement (Equation (1)–(3)) and steel strands (Equations (4)–(6)). Up until now, Equation (1) is still the main equation to calculate the transfer length of CFRP and CFCC reinforcements. There are different types of FRP reinforcement (CFRP, CFCC, GFRP, AFRP, BFRP) with different surface conditions and material properties. Therefore, other researchers proposed values of the empirical coefficient α_t_ for different types of FRP reinforcement (Table 9). The values proposed by [31,32] and adopted in [10,12] are α_t_ = 1.9 and α_t_ = 4.8 for CFRP Leadline bars and CFCC strands respectively. The authors of [39] proposed α_t_ = 1.95 and α_t_ = 2.12 for CFRP Leadline bars and CFCC strands, respectively. In [36,37], α_t_ = 2.6 was proposed for GFRP ribbed bars. Additionally, [43,44] investigated specimens made of self-compacting concrete (SCC) and prestressed with CFRP bars and proposed α_t_ = 2.84 − f_pi_/800.

## 4. Results

### 4.1. Database of Transfer Length Results

A literature review of the experimental results of the transfer lengths of different types of pretensioned FRP reinforcement was performed. In total, 318 specimens were found. Table 10 provides a summary of the initial parameters of the database (Table A1, Table A2, Table A3, Table A4 and Table A5 in Appendix A) of the transfer length of FRP reinforcement. Specifically, 26 of 26, 62 of 99, 73 of 99, 70 of 88, and 6 of 6 specimens prestressed with GFRP (Table A1), CFCC (Table A2), CFRP (Table A3), AFRP (Table A4), and BFRP (Table A5) reinforcement were found with the transfer length results, respectively. Table A1, Table A2, Table A3, Table A4 and Table A5 present the original specimen number from the experimental research, type of FRP reinforcement, surface conditions of FRP reinforcement, specimen type and dimensions (b × h × l—width and height of the cross-section and length of the specimen), release type of prestressed reinforcement, information about the existence of shear reinforcement in the cross-section of the specimen, protective concrete cover (c), reinforcement diameter (Ø), cross-sectional area of one prestressed bar (A_p_), modulus of elasticity (E_p_), tensile strength (f_pu_), initial stresses (f_pi_) of reinforcement, the ratio between initial stresses and tensile strength of reinforcement (f_pi_/f_pu_), concrete compressive strength at transfer (f_ci_), average transfer length (L_t_) of pretensioned reinforcement.

### 4.2. Analysis of Experimental Results

**Figure 1:**
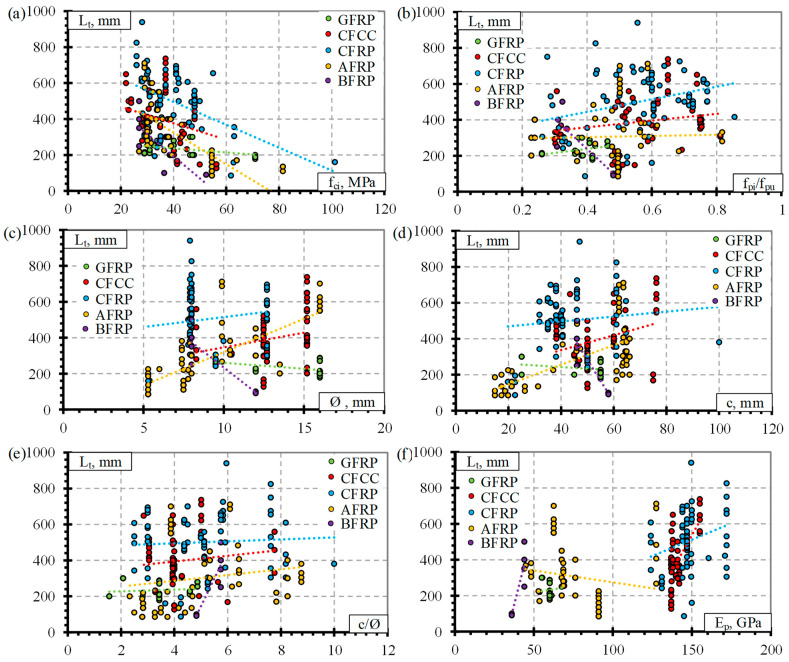
Relationship between transfer length and (**a**) concrete compressive strength at transfer (f_ci_), (**b**) ratio between initial stress and tensile strength (f_pi_/f_pu_), (**c**) reinforcement diameter (Ø), (**d**) concrete cover (c), (**e**) ratio between the concrete cover and reinforcement diameter (c/Ø), (**f**) modulus of elasticity (E_p_) of different types of pretensioned FRP reinforcement.

The experimental results for the transfer length sof different types of FRP reinforcement (GFRP, CFCC, CFRP, AFRP, and BFRP) found in the literature are analyzed in this chapter. Figure 1 shows the distribution of the experimental transfer lengths (L_t_) of different types of pretensioned FRP reinforcement under the influence of different experimental parameters: concrete compressive strength at transfer (f_ci_), the ratio between initial stress in pretensioned reinforcement, and tensile strength (f_pi_/f_pu_), reinforcement diameter (Ø), concrete protective cover (c), the ratio between the concrete cover and reinforcement diameter (c/Ø) and modulus of elasticity of FRP reinforcement (E_p_). The ranges of influential parameters (f_ci_, f_pi_/f_pu_, Ø, c, c/Ø and E_p_) presented in Figure 1 are presented in Table 10.

The results show that the transfer lengths of different types of pretensioned FRP reinforcement decrease with increasing concrete compressive strength at the time of pretensioned reinforcement release (Figure 1a). In general, the increased compressive strength of concrete enhances the bond between the reinforcement and the concrete. Therefore, it provides better anchorage properties for the pretensioned reinforcement. Research by other authors confirms the trend that the transfer length decreases with the increase of concrete strength. However, according to the results of Zou [25] and Zawam with colleagues [26,27], the rate of decrease in transfer length is lower for the AFRP and GFRP bars. Therefore, the concrete strength is more significant for CFCC and CFRP reinforcement.

The transfer length of different types of pretensioned FRP reinforcement tends to increase with an increase in the f_pi_/f_pu_ ratio (Figure 1b). The higher prestress level transfers a higher prestress force to the concrete, which induces greater damage to the bond between the reinforcement and the concrete. Therefore, the transfer length is usually higher for a higher level of prestress. Some results in the literature can be found that show little influence of prestress level on the transfer length. It can be attributed to CFCC [29,30,39] and CFRP [31,32,39,45,46] reinforcement (Table 3). On the other hand, in [31,32] it is stated that the prestress level may have a greater effect on the transfer length of CFCC strands compared with the concrete strength. Despite some discrepancies between the results of the transfer length of CFCC and the CFRP reinforcement of different authors, the main trend of increasing the transfer length with an increase in prestress level remains for different types of FRP reinforcement (GFRP, CFCC, CFRP, AFRP) (Figure 1b).

The transfer lengths of different types of pretensioned FRP reinforcement (CFCC, CFRP, and AFRP) increase with increasing diameter of reinforcement (Figure 1c). For the larger reinforcement diameter, the prestress force will be higher than for the lower reinforcement diameter for the same prestress level. Therefore, higher prestress force transferred to the concrete will cause more damage to the surface between the reinforcement and the concrete. Additionally, this damage will be distributed in a larger contact area with a larger reinforcement diameter. Therefore, the damage to the bond of a larger reinforcement diameter will be larger and will cause a longer transfer length. However, the results of GFRP reinforcement are the opposite (Figure 1c). Therefore, it is evident that reinforcement diameter alone cannot be assessed as the main factor influencing the transfer length.

The transfer lengths of different types of pretensioned FRP reinforcement (CFCC, CFRP, and AFRP) increase with an increase in concrete protective cover (Figure 1d). The release of pretensioned reinforcement induces splitting tensile stresses in concrete due to the Hoyer (wedge) effect, which is the result of the swelling of the reinforcement at transfer. Therefore, the concrete protective cover plays an important role in restricting the splitting of concrete and maintaining sufficient reinforcement bond conditions at transfer. Additional confinement may be introduced in the form of shear stirrups or spiral reinforcement to take over splitting tensile stresses. As in the case of reinforcement diameter (Figure 1c), the transfer length of the GFRP reinforcement decreases with increasing concrete cover (Figure 1d). Therefore, it shows that the transfer length should be compared by evaluating the combination of several influencing factors. Additionally, the concrete cover is more important in the case of durability and splitting of the concrete anchorage zone at transfer.

As in the case of reinforcement diameter (Figure 1c) and concrete cover (Figure 1d), the transfer lengths of different types of pretensioned FRP reinforcement increase with increasing ratio c/Ø (Figure 1e). It should be noted that the increase in the ratio c/Ø with an increase in transfer length is significantly reduced compared to the results presented in Figure 1c,d. Therefore, the evaluation of these two parameters (c and Ø) provides a more uniform distribution of the relationship between the transfer length and c/Ø. Additionally, Taerwe and Pallemans [35] determined that for sand-coated AFRP bars the transfer length decreased with an increase of c/Ø up to approximately 3.5. The further increase in concrete cover did not have a significant influence on the transfer length of the AFRP bars. Therefore, it shows the importance of choosing the appropriate value of the concrete cover (c) for a certain reinforcement diameter (Ø). Additionally, it is again proven that the transfer length should be evaluated in a combination of several influencing factors. The ratio c/Ø is important to ensure the crack-free introduction of the prestressing force into the concrete, while the absolute concrete protective cover is important for the durability of the member.

A comparison of the transfer length and modulus of elasticity of different types of FRP reinforcement shows that in most cases, the transfer length of FRP reinforcement (GFRP, CFCC, and CFRP) increases with increasing modulus of elasticity (Figure 1f). In [36,51,52], it was stated that a lower modulus of elasticity will cause a greater swelling of the reinforcement, resulting in an increase in the confining stresses at transfer. It improves the bond strength of the reinforcement, resulting in a shorter transfer length [36]. The results of [33,47,53,54] showed that the transfer length of AFRP bars was lower than that of the CFRP Leadline bar due to the lower modulus of elasticity of the AFRP reinforcement. However, Figure 1f shows that the transfer length of AFRP bars is slightly decreasing with increasing modulus of elasticity. This may be related to the higher range of modulus of elasticity of AFRP reinforcement (Table 10) and the additional variation of other parameters (nonidentical).

The transfer length of the BFRP bar also decreases with an increase in concrete strength (Figure 1a). However, the trends of other parameters (f_pi_/f_pu_, Ø, c, c/Ø, and E_p_) with respect to the transfer length are opposite compared to GFRP, CFCC, CFRP, and AFRP reinforcements. The results of only six specimens pretensioned with BFRP reinforcement are presented in the database of transfer length (Table A5). The number of specimens and the variation of the parameters are too small. Therefore, it is difficult to distinguish a clear influence of different parameters on the transfer length of BFRP bars. Therefore, broader experimental research needs to be performed for a better understanding the behavior of anchorage zone of prestressed BFRP bar.

**Figure 2:**
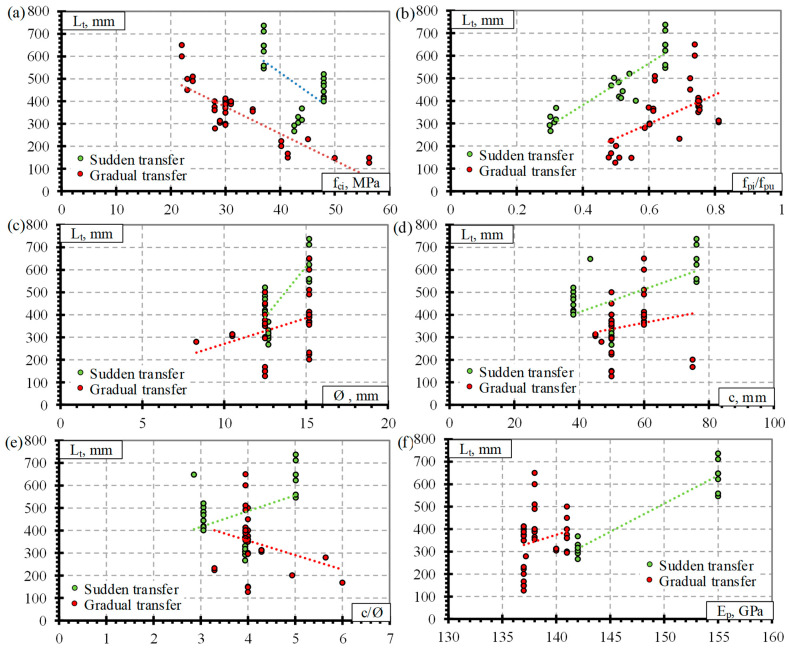
Relationship between transfer length and (**a**) concrete compressive strength at transfer (f_ci_), (**b**) ratio between initial stress and tensile strength (f_pi_/f_pu_), (**c**) reinforcement diameter (Ø), (**d**) concrete cover (c), (**e**) ratio between the concrete cover and reinforcement diameter (c/Ø), (**f**) modulus of elasticity (E_p_) of different types of CFCC pretensioned reinforcement under different methods of prestress transfer.

The prestress force can be transferred to the concrete in two ways: gradually or suddenly. Therefore, the influence of the type of release of CFCC reinforcement (sudden or gradual) on the transfer length and parameters (f_ci_, f_pi_/f_pu_, Ø, c, c/Ø and E_p_) influencing the transfer length is presented in Figure 2. It can be seen that there is a clear difference between the results, and in most cases, a sudden release gives higher transfer length results compared to a gradual release of CFCC strands. The same tendency is observed with pretensioned steel strands. The influence of reinforcement release type on the transfer length of CFCC strand can be affected by the the similar shape of the the CFCC and steel strand (seven helically wounded wires).

### 4.3. Derivation of the Coefficient α_t_

**Figure 3:**
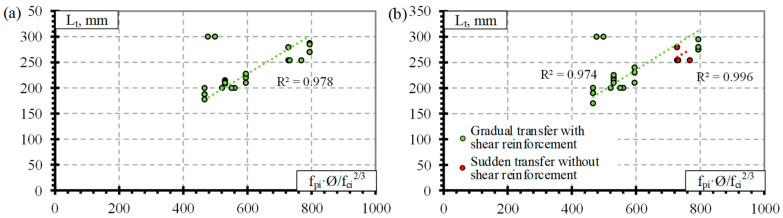
Relationship between the transfer length of GFRP reinforcement and $f_{pi}\cdot Ø/f_{ci}^{2/3}$: (**a**) all results, (**b**) effect of the shear reinforcement and the type of transfer of the prestress.

**Figure 4:**
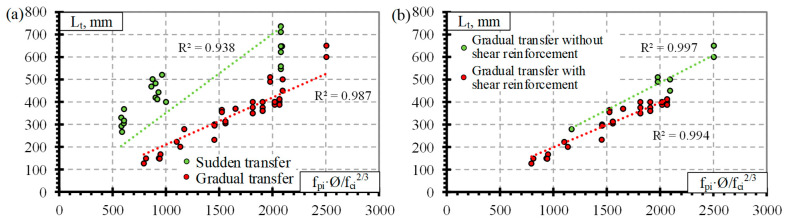
Relationship between the transfer length of CFCC strands and $f_{pi}\cdot Ø/f_{ci}^{2/3}$: (**a**) effect of types of transfer of prestressing, (**b**) effect of shear reinforcement.

**Figure 5:**
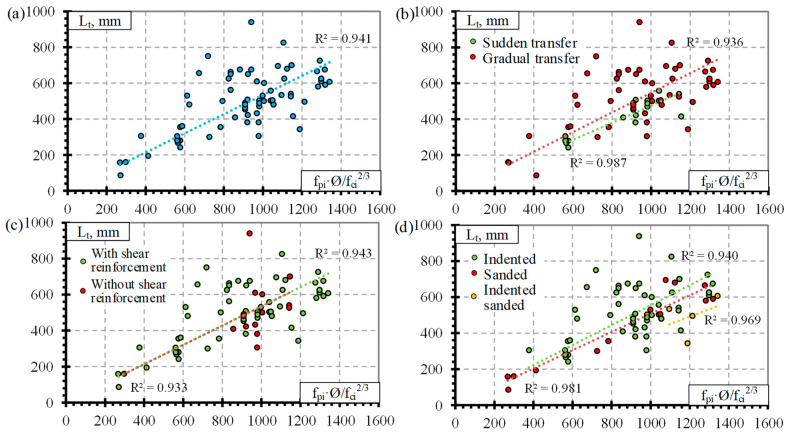
Relationship between transfer length of CFRP bars and $f_{pi}\cdot Ø/f_{ci}^{2/3}$: (**a**) all results, (**b**) effect of type of prestressing transfer, (**c**) effect of shear reinforcement, (**d**) effect of reinforcement surface conditions.

**Figure 6:**
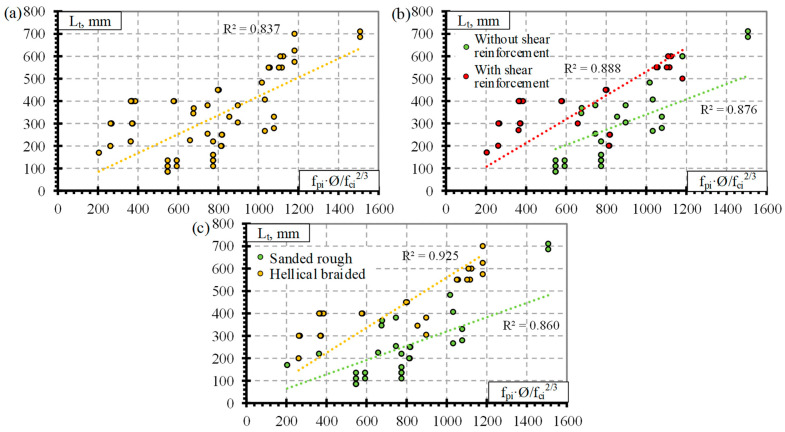
Relationship between transfer length of AFRF bars and $f_{pi}\cdot Ø/f_{ci}^{2/3}$: (**a**) all results, (**b**) effect of shear reinforcement, (**c**) effect of reinforcement surface conditions.

Previous analysis of the results (Figure 1 and Figure 2) showed that the influence of separate factors on the transfer length of prestressed FRP reinforcement does not always provide a qualitative comparison. Equation (1) provided in Table 8 is proposed for prestressed FRP reinforcement and takes into account initial prestress (f_pi_), reinforcement diameter (Ø), and concrete compressive strength at transfer (f_ci_). Therefore, for a better analysis of the results based on Equation (1), a graphical comparison of the transfer length of different FRP reinforcements (GFRP, CFCC, CFRP, and AFRP) and $f_{pi}\cdot Ø/f_{ci}^{2/3}$ is presented in Figure 3, Figure 4, Figure 5 and Figure 6. Additionally, these graphs represent a distribution of the results with the proposed average values for the coefficient α_t_.

It was calculated that for the database of GFRP reinforcement, the average coefficient α_t_ is 2.58 with the standard deviation (STD) of 0.33 and the coefficient of variation (COV) of 12.8% (Table 8) (for the concrete strength 29–71 MPa, the level of prestressing 26–47% and the diameter of the reinforcement 9.5–16 mm). The graphical representation is presented in Figure 3a with a very strong correlation coefficient R^2^ = 0.98. Almost all GFRP bars had ribbed surfaces, and the influence of the reinforcement surface conditions could not be determined. Additionally, Figure 3b shows that the influence of shear reinforcement and the type of prestressed reinforcement release (sudden or gradual) has an almost negligible effect on the transfer length of the GFRP reinforcement. However, it should be mentioned that the database of transfer length results of GFRP reinforcement is quite small (Table A1) compared with other FRP types (Table A2, Table A3 and Table A4): only five specimens reinforced with shear reinforcement and prestressed with GFRP reinforcement affected by a sudden transfer of prestressing were found in the literature. Therefore, more research is needed to investigate the effect of shear reinforcement and the type of release of prestressed GFRP reinforcement on the transfer length.

The type of CFCC strand release has already been shown to affect the transfer length even when the influence of separate factors was analyzed (Figure 2). Furthermore, the effect is more obvious when comparing the transfer length with $f_{pi}\cdot Ø/f_{ci}^{2/3}$ (Figure 4a). Figure 4a represents the distribution of the results corresponding to an average α_t_ = 4.99 with STD = 0.62, COV = 12.5%, and R^2^ = 0.99 for the gradual transfer of prestress (for concrete strength of 22–56 MPa, level of prestress 31–81% and diameter of reinforcement 8.3–15.2 mm) and α_t_ = 2.42 with STD = 0.67, COV = 27.7%, and R^2^ = 0.94 for sudden transfer of prestress (Table 11) (for concrete strength of 37–48 MPa, level of prestressing 30–65% and diameter of reinforcement 12.5–15.2 mm). Additionally, the higher variation of the results (27.7% and 12.5% for sudden and gradual release, respectively) of the sudden type of reinforcement release shows that this type of release is more dangerous and can cause more damage to the anchorage zone of prestressed CFCC strands and, therefore, increase the transfer length. According to Figure 4b, there is no clear influence of shear reinforcement on the transfer length of the CFCC strand. All CFCC seven-wire strands in the collected database have helical plain or almost plain surfaces, and therefore, their effect on the transfer length could not be determined.

Figure 5a presents the transfer length distribution of CFRP bars with respect to $f_{pi}\cdot Ø/f_{ci}^{2/3}$ with an average α_t_ = 1.92 with STD = 0.48, COV = 24.8%, and R^2^ = 0.94 (Table 11) (for concrete strength of 26–101 Mpa, prestress level 26–86%, and reinforcement diameter 5.3–12.7 mm). According to Figure 5b,c, there is no influence of the type of transfer of prestressing and shear reinforcement on the transfer length of CFRP bars. Most CFRP bars were Leadline bars with a spirally indented surface (Table A3), and a few studies were performed on spirally indented, sanded, and spirally indented and sanded CFRP bars. Khin et al. [50] determined that CFRP reinforcement with spirally indented or sanded surface had up to 25% lower transfer length compared with CFRP reinforcement with a helical plain surface. The trend lines show that the higher the roughness of the CFRP bar surface, the lower the transfer length (Figure 5d). However, the scatter of the bigger database results is too large to qualitatively confirm this trend.

Figure 6a presents the transfer length distribution of AFRP bars with respect to $f_{pi}\cdot Ø/f_{ci}^{2/3}$ with an average α_t_ = 2.9 with STD = 1.8, COV = 62.1%, and R^2^ = 0.84 (Table 11) (for concrete strength of 27–81 MPa, prestressing level 23–82% and reinforcement diameter 5.3–16 mm). The variation in the results is very high (62%). Therefore, the additional influence of other variables was investigated on the transfer length of the AFRP bars. According to Figure 6b, shear reinforcement may have some influence on the transfer length of AFRP bars. However, since the transfer length is shown to be higher for specimens with shear reinforcement, it contradicts the results for GFRP (Figure 3b) and CFCC (Figure 4b) reinforcement. Therefore, it was decided that the influence of shear reinforcement will be neglected in determining the coefficient α_t_. Additionally, Figure 6c shows that two reinforcement surface conditions can be distinguished for the AFRP bars. Therefore, the transfer length is higher for smooth braided AFRP bars compared to sanded and rough FRP bars. The same trend of decrease in transfer length with an increase in reinforcement surface roughness was observed by [24,35,38,47,48]. Additionally, the ribbed surface of the GFRP bar [36,37] and the sand-coated surface of the AFRP bar [24,38] gave similar transfer length results; the increased roughness of reinforcement increases the bond between reinforcement and concrete and therefore the transfer length. Furthermore, reinforcement classification according to surface roughness gives different results of α_t_ = 1.53 with STD = 0.55, COV = 35.8%, and R^2^ = 0.93 for smooth braided AFRP bars (for concrete strength of 29–39 MPa, prestressing level 23–58%, and reinforcement diameter 8–16 mm) and α_t_ = 3.99 with STD = 1.70, COV = 42.7%, and R^2^ = 0.86 for sanded and rough AFRP bars (Table 11) (for concrete strength of 27–81 MPa, prestressing level 37–82%, and reinforcement diameter 5.3–13.5 mm). These results show that an additional classification of AFRP bars according to surface conditions reduces the variation of the results from 62% to 36%–43%. However, this level of variation in the results is still high compared with GFRP, CFCC, and CFRP reinforcement. Therefore, the surface condition of AFRP bars should be evaluated in additional comprehensive research.

**Figure 7:**
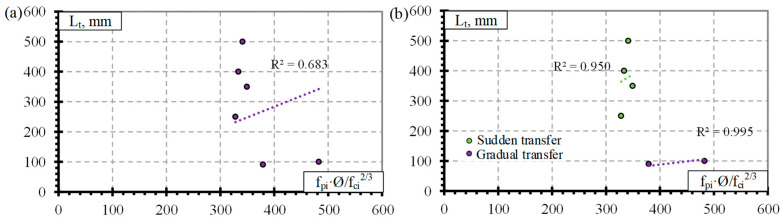
Relationship between the transfer length of BFRP reinforcement and $f_{pi}\cdot Ø/f_{ci}^{2/3}$: (**a**) all results, (**b**) effect of the type of transfer of the prestressing.

Figure 7a presents the transfer length distribution of the BFRP bars with respect to $f_{pi}\cdot Ø/f_{ci}^{2/3}$ with an average α_t_ = 2.1 with STD = 1.7, COV = 79.2%, and R^2^ = 0.68 (Table 11) (for concrete strength of 27–52.4 MPa, prestressing level 34–48%, and reinforcement diameter 8–12 mm). The low number of results gives high variation in α_t_. Therefore, it cannot be applied to calculate the transfer length of BFRP bars. Additionally, Figure 7b shows a preliminary comparison of the transfer length and $f_{pi}\cdot Ø/f_{ci}^{2/3}$ with the influence of release type of reinforcement. The results show that sudden transfer of prestress induces a longer transfer length of the BFRP bar compared to gradual transfer of prestress (Figure 7b). This confirms the results of the CFCC strands influenced by different types of reinforcement release (sudden or gradual) (Figure 4a). Therefore, the influence of the type of BFRP reinforcement release and other important factors (f_ci_, f_pi_/f_pu_, c) on the transfer length could be the topic of future research.

### 4.4. Comparison of Experimental and Theoretical Results

**Figure 8:**
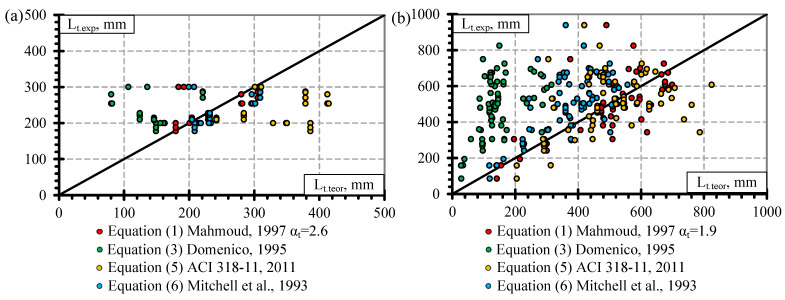
Relationship between the experimental and theoretical transfer lengths of (**a**) GFRP reinforcement and (**b**) CFRP reinforcement [30,31,63,64].

**Figure 9:**
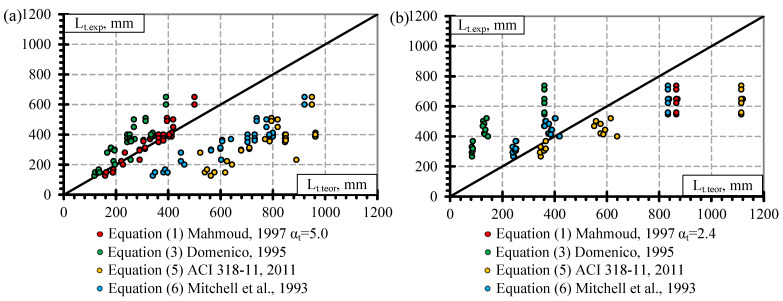
Relationship between experimental and theoretical transfer lengths of CFCC strands (**a**) for the gradual transfer of prestress, (**b**) for sudden transfer of prestress [30,31,63,64].

**Figure 10:**
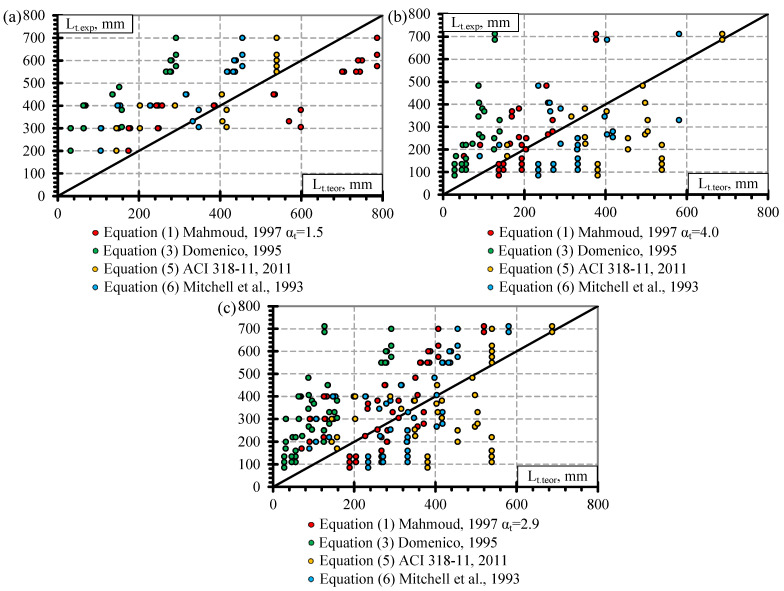
Relationship between the experimental and theoretical transfer lengths of AFRP reinforcement with different surface conditions: (**a**) smooth braided, (**b**) sanded and rough, (**c**) both (all results) [30,31,63,64].

In this section, theoretical models for the calculation of transfer length (Table 8, Section 3) are compared with experimental results from the literature (Table A1, Table A2, Table A3 and Table A4). In particular, models are presented by [10,12,31,32] (Equation (1)), [63] Equation (6), [64] Equation (5), and [30] Equation (3). Equation (2) proposed by Zou [33] is not analyzed due to the lack of correlation with the experimental results found in the literature. Furthermore, Equation (4) is not taken into account because it is proposed for steel strands [70] and is very similar to Equation (1) proposed for FRP reinforcement. The results presented in Figure 8, Figure 9 and Figure 10 show the relationships between the experimental and theoretical results of the transfer length of different FRP reinforcements. In addition, they show the differences between different theoretical models for calculating the transfer length. The theoretical model proposed by [31,32] is presented in Figure 8, Figure 9 and Figure 10 with the α_t_ coefficients proposed in this article (Table 8).

From Figure 8a, it is clear that the theoretical models presented by [30,64] are less accurate than the models presented by [31,32,63]. Furthermore, the [64] model overestimates experimental results on average by 47% (L_t,teor_/L_t.exp_ = 1.47) with STD = 0.32 and COV = 21.7% and [30] underestimates experimental results of the transfer length of prestressed GFRP reinforcement on average by 38% (L_t,teor_/L_t.exp_ = 0.62) with STD = 0.19 and COV = 31.2%. The models of Mitchell [63] and Mahmoud [31,32] (α_t_ = 2.6) show similar results with L_t,teor_/L_t.exp_ = 1.05, STD = 0.13, COV = 12.1% and with L_t,teor_/L_t.exp_ = 1.0 STD = 0.13, COV = 12.6%, respectively. The models presented by [31,32,63] are in the best agreement with the experimental results (difference up to 5%) and the variation of the results is the lowest (COV = 12%). Therefore, it shows the best agreement between theoretical and experimental results.

In the case of CFRP reinforcement (Figure 8b), Equation (3) gives the most inappropriate results with an underestimation of the experimental results by 70% (L_t,teor_/L_t.exp_ = 0.30) with STD = 0.12, COV = 40.2%. Equation (6) also underestimates the experimental results with L_t,teor_/L_t.exp_ = 0.82, STD = 0.28, COV = 34%, but Equation (5) overestimates the results with L_t,teor_/L_t.exp_ = 1.17, STD = 0.55, COV = 47.2%. Despite the relatively high variation of the results the most accurate prediction of experimental results was received by Equation (1) (α_t_ = 1.9) with L_t,teor_/L_t.exp_ = 1.01, STD = 0.33, COV = 32.5%.

Figure 9 distinguishes the comparison of experimental and theoretical results of the transfer length of CFCC strands for the gradual (Figure 9a) and sudden (Figure 9b) types of prestressing release. Equation (5) significantly overestimates the experimental results for gradual transfer of prestress with L_t,teor_/L_t.exp_ = 2.31, STD = 0.81, COV = 35% (Figure 9a). Additionally, Equation (5) gives the same trend of the results for sudden transfer of prestressing with L_t,teor_/L_t.exp_ = 1.41, STD = 0.30, COV = 21.3% (Figure 9b). However, Equation (3) significantly underestimates the experimental results for both gradual (Figure 9a) and sudden (Figure 9b) prestress transfer with L_t,teor_/L_t.exp_ = 0.69, STD = 0.19, COV = 27.2% and L_t,teor_/L_t.exp_ = 0.38, STD = 0.14, COV = 37%, respectively. Despite the low precision (L_t,teor_/L_t.exp_ = 1.84, STD = 0.48, COV = 25.9%) in predicting the transfer length for the gradual transfer of prestress (Figure 9a), Equation (6) presents a very accurate prediction of the experimental results for the sudden transfer of prestress (Figure 9b) with L_t,teor_/L_t.exp_ = 0.99, STD = 0.26, COV = 26%. Additionally, Equation (1) provides a very accurate prediction of the transfer length for gradual (α_t_ = 5.0) (Figure 9a) and sudden (α_t_ = 2.4) (Figure 9b) types of release with L_t,teor_/L_t.exp_ = 1.0, STD = 0.12, COV = 12.5% and L_t,teor_/L_t.exp_ = 1.02, STD = 0.27, COV = 26.6%, respectively. The higher variation in the results for sudden prestress transfer shows that it has more damage to the bond of reinforcement and results in a longer transfer length. Part of the results of Equation (6) coincide with the results of Equation (1) for sudden transfer of prestress. It shows that the combination of empirical coefficient (20.7) and $f_{ci}^{1/2}$ in Equation (6) is similar to the combination of coefficient α_t_ = 2.4 and $f_{ci}^{2/3}$ in Equation (1) in case of sudden transfer of prestress. However, Equation (1) provides more consistent results for both gradual and sudden transfer of prestress.

Figure 10 presents the comparison of the experimental and theoretical results of the transfer length of AFRP reinforcement with smooth braided (Figure 10a) and sanded and rough (Figure 10b) surfaces. Theoretical results calculated according to Equations (3), (5), and (6) give lower transfer lengths of AFRP reinforcement with a smooth braided surface compared to the experimental results with L_t,teor_/L_t.exp_ = 0.3, STD = 0.15, COV = 51.8%; L_t,teor_/L_t.exp_ = 0.8, STD = 0.23, COV = 30.1%; and L_t,teor_/L_t.exp_ = 0.6, STD = 0.21, COV = 34.3%, respectively (Figure 10a). However, the prediction of Equation (1) (with α_t_ = 1.5) is closer to the experimental results with L_t,teor_/L_t.exp_ = 1.02, STD = 0.36, COV = 35.8%, and more values are on the safe side (Figure 10a). The prediction of the transfer length of AFRP reinforcement with sanded and rough surfaces (Figure 10b) was also significantly underestimated according to Equation (3) with L_t,teor_/L_t.exp_ = 0.3, STD = 0.12, COV = 36.6%. However, in this case, Equations (5) and (6) significantly overestimate the transfer length with L_t,teor_/L_t.exp_ = 2.9, STD = 1.4, COV = 48.9% and L_t,teor_/L_t.exp_ = 1.8, STD = 0.77, COV = 42.3%, respectively. A similar trend can be observed for all results from the database (without grouping according to surface conditions) (Figure 10c) calculated according to Equations (3), (5) and (6) with L_t,teor_/L_t.exp_ = 0.3, STD = 0.14, COV = 42.6%; L_t,teor_/L_t.exp_ = 1.8, STD = 1.41, COV = 78.6%; and L_t,teor_/L_t.exp_ = 1.2, STD = 0.79, COV = 66%, respectively. The most accurate prediction of the results was obtained according to Equation (1) for AFRP reinforcement with sanded and rough surfaces (α_t_ = 4.0) (Figure 10b) and all types of AFRP reinforcement (α_t_ = 2.9) (Figure 10c) with L_t,teor_/L_t.exp_ = 1.0, STD = 0.43, COV = 42.7% and L_t,teor_/L_t.exp_ = 1.0, STD = 0.62, COV = 62.1%, respectively. However, the variation in the results is still very high for AFRP reinforcement.

Different values of coefficient α_t_ (used in Equation (1)) were proposed by other authors (Table 9) and calculated in this article (see Section 4.3 and Table 11). The results in this article confirm the α_t_ = 2.6 proposed by [36,37] for calculating the transfer length of the GFRP bars. Additionally, the α_t_ = 1.92 calculated in this article is in close agreement with the proposals of other authors [10,12,31,32] α_t_ = 1.90 and [39] α_t_ = 1.95 for CFRP bars. The proposed α_t_ = 5.0 for gradually released CFRP bars is slightly higher than α_t_ = 4.8 [10,12,31,32] and significantly higher than α_t_ = 2.12 [36,37]. The suggested α_t_ = 2.4 is new, as there is no proposal of this coefficient for the sudden release of pretensioned CFCC reinforcement in the literature. This value is between the α_t_ = 4.8 [10,12,31,32] and α_t_ = 2.12 [36,37] suggested in the literature for the gradual release of pretensioned CFCC reinforcement. Furthermore, the literature review does not provide any proposal of α_t_ for the AFRP reinforcement. Therefore, three possible values of α_t_ were proposed in this article: α_t_ = 2.9 for all different types of AFRP reinforcement, α_t_ = 1.5 for smooth braided AFRP bars, and α_t_ = 4.0 for sanded and rough AFRP bars. However, the high variation in the transfer length results of AFRP reinforcement suggests additional research and analysis for better understanding its behavior.

**Figure 11:**
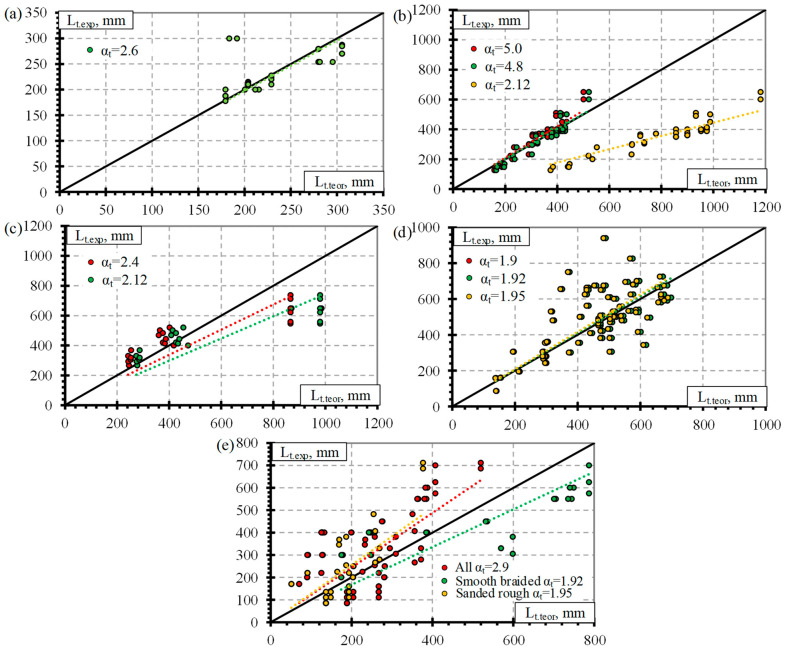
Comparison of experimental and theoretical transfer length results: (**a**) GFRP reinforcement, (**b**) CFCC strands with gradual transfer, (**c**) CFCC strands with sudden transfer, (**d**) CFRP bars, (**e**) AFRP bars.

The comparison of the ratio between theoretical and experimental results of the transfer length (L_t.teor_/L_t.exp_) of different types of FRP reinforcement for different values of α_t_ is presented in Figure 11. Table 12 presents an additional statistical analysis.

The results for the GFRP bars with α_t_ = 2.6 (Table 12 and Figure 11a) show a low COV = 12.6% and a high inbound value (between the upper and lower bounds) of 80%. Therefore, the proposed coefficient is suitable for GFRP bars.

The α_t_ = 2.12 proposed by [39] for the gradual release of CFCC strands is not suitable due to a very high overestimation of the experimental transfer length with a mean L_t.teor_/L_t.exp_ = 2.35, and all 41 specimens are on the safe side (Table 12 and Figure 11b). Furthermore, more CFCC strands with α_t_ = 4.8 are on the safe side compared with α_t_ = 5.0 (Table 12), and considering that all other statistical parameters are almost identical, α_t_ = 4.8 may be a more appropriate choice for gradual transfer. For sudden CFCC strand release, the proposed α_t_ = 2.4 gives better agreement with the experimental results for transfer length with theoretical values higher by 1% compared with results higher by 14% with α_t_ = 2.12 (Table 12). Additionally, the linear trend line presented in Figure 11c shows that the theoretical results with α_t_ = 2.4 are in closer agreement with the experimental transfer length.

The results for the relationship between the theoretical and experimental transfer lengths are almost identical for CFRP bars, with α_t_ ranging from 1.90 to 1.95 (Table 12 and Figure 11d). For α_t_ = 1.95, the theoretical results on average are 2% lower than the experimental ones. Therefore, it is better to use α_t_ = 1.9 for determining the transfer length of the CFRP bars.

The proposed α_t_ = 2.9 for the AFRP bars provides 65% inbound values (Table 12 and Figure 11e). However, the 62% variation in the results is too large. Additionally, a separate α_t_ = 1.5 for FRP bars with the smooth braided surface was proposed and provided a lower variation of the results (35%) and a similar inbound 61%. The α_t_ = 4.0 proposed for the FRP reinforcement with sanded and rough surface shows a higher variation in the theoretical results (42%) compared with the results of the smooth braided AFRP bars (35%), but less than the results for all AFRP reinforcement with α_t_ = 2.9. As the results of the sanded and rough AFRP bars provide about 49% inbound values, the amount of overestimated (OV = 20) and underestimated (UV = 19) values is similar.

## 5. Conclusions

A large database of the transfer lengths of different FRP reinforcements was collected, and the analysis of experimental results, description of theoretical models, and comparison of experimental and theoretical results led to the following conclusions and proposals:

1. The analysis of the literature showed that the concrete compressive strength has a greater influence on the transfer lengths of CFRP and CFCC reinforcement, and the prestress level has a greater effect on the transfer lengths of AFRP and GFRP reinforcement. Additionally, most transfer lengths of FRP reinforcements (GFRP, CFCC, CFRP, and AFRP) are directly proportional to prestress level, reinforcement diameter, and modulus of elasticity of reinforcement and inversely proportional to concrete strength.
2. The minimum concrete cover to avoid concrete splitting in the anchorage zone of the FRP reinforcement is 1.9·Ø for the CFRP bar and 2.8·Ø for the AFRP and GFRP bars. Additional research is needed for the CFCC strand, but the sufficient concrete cover for the CFCC strand is 4·Ø. Therefore, the concrete cover of 3.5·Ø and above does not have a significant influence on the transfer length of FRP reinforcement.
3. Equation (1) was determined to give the most accurate prediction of the transfer length by applying different values of coefficient α_t_ for different types of FRP reinforcement. The results of this article confirmed that the coefficients α_t_ = 2.6, α_t_ = 1.9, and α_t_ = 4.8 proposed by other authors are suitable for predicting the transfer length of the GFRP bars, CFRP bars, and CFCC strands (only gradual type of release), respectively.
4. The analysis of the database revealed that the transfer length of CFCC strands is highly dependent on the type of prestress transfer. Therefore, a new coefficient α_t_ = 2.4 was proposed to determine the transfer length of the suddenly released CFCC strand based on a collected database of the transfer length results. The proposed value is valid for a concrete strength of 37–48 MPa, prestress level 30–65%, and reinforcement diameter of 12.5–15.2 mm.
5. It was determined that there is a correlation between the surface conditions and the transfer length of AFRP bars. Despite the relatively high variation in the results, in this article, new values are proposed: α_t_ = 1.5 for smooth braided AFRP bars (for concrete strength 29–39 MPa, prestress level 23–58%, and reinforcement diameter 8–16 mm) and α_t_ = 4.0 for sanded and rough AFRP bars (for concrete strength of 27–81 MPa, prestress level 37–82%, and reinforcement diameter 5.3–13.5 mm).
6. The analysis of the transfer length and the new values proposed for the coefficient α_t_ provides possibilities for adapting it to design codes for engineering applications and performing additional research that fills the missing gaps in the field. In particular, additional research is needed on the effects of shear reinforcement, release type of the prestressed GFRP and AFRP reinforcements, and the surface conditions of the AFRP and CFRP bars. Furthermore, the influence of prestressing transfer method and other important parameters (f_ci_, f_pi_/f_pu_, Ø, c) on the transfer length of the BFRP reinforcement could be a subject of future research.

## Figures and Tables

**Table 1:** Basic physical and mechanical properties of different FRP bars [10,11,12,13,14].

FRP Type	Density, kg/m^3^	Tensile Strength, MPa	Modulus of Elasticity, GPa	Elongation, %	Longitudinal Coefficient of Linear Expansion (10^−6^/°C)
GFRP	1250–2500	480–1600	35–86	1.2–5.0	6.0–10.0
CFRP	1500–2100	600–3920	120–784	0.5–1.9	−9.0–0.0
AFRP	1250–1450	1720–3620	41–175	1.4–4.4	−6.0–2.0
BFRP	1900–2100	600–1650	45–65	1.2–3.0	9.0–12.0
Prestressing steel	7850	1770–2060	195–205	3.5–4.0	12.0

**Table 2:** Summary of transfer length results on the influence of concrete compressive strength at transfer.

Reference	FRP Type	FRP Surface	c, mm	Ø, mm	c/Ø	E_p_, GPa	f_pi_/f_pu_	f_ci.min_, MPa	f_ci.max_, MPa	L_t.max_, mm	L_t.min_, mm
[31,32]	CFCC	Helical plain	50	12.5	4.0	141	0.75	23	28	475	374
	CFCC	Helical plain	60	15.2	3.9	138	0.6	24	35	500	360
	CFCC	Helical plain	60	15.2	3.9	138	0.75	22	31	625	395
[29,30]	CFCC	Helical plain	50	12.5	4.0	137	0.5	41	56	158.5	137.5
[31,32]	CFRP	Spirally indented	35	8.0	4.4	147	0.75	28	35	620	480
[33]	CFRP	Spirally indented	61	8.0	7.6	172	0.3	26	63	750	305
	CFRP	Spirally indented	61	8.0	7.6	172	0.4	26	63	825	355
[34]	CFRP	Spirally indented sanded	22.5	5.3	4.2	509	0.52	33	63	194	158
[33]	AFRP	Sanded	61.1	7.8	7.8	54	0.4	27	64	220	170
[35,48]	AFRP	Sanded	21.3	7.5	2.8	91	0.5	54.5	81.5	169	135
[36,37]	GFRP	Ribbed	55	16.0	3.4	60	0.4	31	71	278	188
[55]	BFRP	Sanded	58	12.0	4.8	36	0.48	36.5	52.4	100	90

c—concrete cover, Ø—reinforcement diameter, E_p_—modulus of elasticity of reinforcement, f_pi_—initial prestress of reinforcement, f_pu_—tensile strength of reinforcement, f_ci.min_ and f_ci.max_—lower and higher concrete strength, respectively, L_t.min_ and L_t.max_—lower and higher transfer length, respectively.

**Table 3:** Summary of transfer length results for the influence of prestress level of FRP reinforcement.

Reference	FRP Type	FRP Surface	c, mm	Ø, mm	c/Ø	E_p_, GPa	f_ci_, MPa	f_pi_/f_pu_ (min)	f_pi_/f_pu_ (max)	L_t.min_, mm	L_t.max_, mm
[39]	CFCC	Helical plain	38.2	12.5	3.1	-	48	0.49	0.56	468	400
[31,32]	CFCC	Helical plain	50	12.5	4.0	141-	28–30	0.6	0.75	298	374
	CFCC	Helical plain	60	15.2	3.9	138	22–24	0.62	0.74	500	625
[29,30]	CFCC	Helical plain	60	15.2	3.9	137	30	0.6	0.75	370	403
[40,41]	CFRP	Spirally indented	46.1	7.8	5.9	147	50	0.5	0.7	360	500
[43,44]	CFRP	Sanded	38.1	12.7	3.0	144	37	0.33	0.62	300	585
	CFRP	Sanded	38.1	12.7	3.0	144	30	0.31	0.56	355	665
[33]	CFRP	Spirally indented	61	8.0	7.6	172	26	0.3	0.4	750	825
	CFRP	Spirally indented	61	8.0	7.6	172	36	0.3	0.4	505	675
	CFRP	Spirally indented	61	8.0	7.6	172	63	0.3	0.4	305	355
[45]	CFRP	Spirally indented sanded	31.8	12.7	2.5	124	46	0.5	0.55	533	495
	CFRP	Spirally indented sanded	31.8	12.7	2.5	124	46	0.5	0.59	533	607
[46]	CFRP	Spirally indented	46.1	7.9	5.8	150	30	0.5	0.6	662	625
	CFRP	Spirally indented	46.1	7.9	5.8	150	30	0.5	0.7	662	700
[39]	CFRP	Spirally indented	40.5	7.9	5.1	147	48	0.6	0.68	470	507
	CFRP	Spirally indented	40.5	7.9	5.1	147	48	0.6	0.75	470	504
[31,32]	CFRP	Spirally indented	35	8.0	4.4	147	34–35	0.61	0.72	458	480
[47]	AFRP	Plain	63.7	9.9	6.4	128	29–31	0.43	0.59	385	699
[36,37]	GFRP	Ribbed	55	16.0	3.4	60	29–31	0.26	0.41	211	278

c—concrete cover, Ø—reinforcement diameter, Ep modulus of elasticity of reinforcement, f_ci_—concrete compressive strength at transfer, f_pi_—initial prestress of reinforcement, f_pu_—tensile strength of reinforcement, f_pi_/f_pu_ (min) and f_pi_/f_pu_ (max)—lower and higher prestress levels, respectively, L_t.min_ and L_t.max_—lower and higher transfer lengths, respectively.

**Table 4:** Summary of the transfer length results for the influence of reinforcement diameter of the FRP reinforcement.

Reference	FRP Type	FRP Surface	c, mm	E_p_, GPa	f_pi_/f_pu_	f_ci_, MPa	Ø_min_, mm	Ø_maz_, mm	L_t.min_, mm	L_t.max_, mm
[29,30]	CFCC	Helical plain	50	137	0.5	40	12.5	15.2	158	212
	CFCC	Helical plain	50	137	0.75	30	12.5	15.2	369	403
[31,32]	CFCC	Helical plain	50	138–141	0.75	22–23	12.5	15.2	475	625
[26]	CFCC	Helical indented	18.8–21	155	0.55	63–69	7.5	7.5	211	211
	CFRP	Spirally indented	15–25	167	0.54	66–68	5.0	5.0	122	122
[24,38]	AFRP	Smooth braided	64	68–76	0.24	36–39	8.0	12.0	260	360
	AFRP	Smooth braided	62	63–76	0.5	31–34	8.0	16.0	400	567
[35,48]	AFRP	Sanded	-	91	0.5	54	5.3	7.5	98	149
[36,37]	GFRP	Ribbed	55	60	0.4	31	12.0	16.0	224	276

c—concrete cover, E_p_—modulus of elasticity of reinforcement, f_ci_—concrete compressive strength at transfer, fpi initial prestress of reinforcement, f_pu_— tensile strength of reinforcement, Ø_min_ and Ø_max_—lower and higher reinforcement diameters, respectively, L_t.min_ and L_t.max_—lower and higher transfer lengths, respectively.

**Table 5:** Summary of transfer length results under the influence of shear reinforcement.

Reference	FRP Type	FRP Surface	c, mm	Ø, mm	c/Ø	E_p_, GPa	f_pi_/f_pu_	f_ci_, MPa	L_t._, mm (With Stirrups)	L_t._, mm (Without Stirrups)
[31,32]	CFCC	Helical plain	50	12.5	4.0	141	0.73–0.75	23–28	374	475
	CFRP	Spirally indented	35	8.0	4.4	147	0.61	34	457	485

c—concrete cover, Ø—reinforcement diameter, E_p_—modulus of elasticity of reinforcement, f_pi_—initial prestress of reinforcement, f_pu_—tensile strength of reinforcement, f_ci_—concrete compressive strength at release, L_t_ (with stirrups)—transfer length of specimen with shear reinforcement, L_t_ (without stirrups)—transfer length of specimen without shear reinforcement.

**Table 6:** Summary of transfer length results on the influence of reinforcement surface conditions.

Reference	FRP Type	FRP Surface	c, mm	Ø, mm	c/Ø	E_p_, GPa	f_pi_/f_pu_	f_ci_, MPa	L_t._, mm
[47]	AFRP	Rough	47.3	7.4	6.4	69	0.51–0.57	30.1	327
	AFRP	Smooth braided	45.8	10.4	4.4	48	0.56–0.58	30	339
	AFRP	Smooth	46.1	9.9	4.7	128	0.42–0.43	31.5	385
[35,48]	AFRP	Sanded	14.9–19.9	5.3	2.8–3.7	91	0.5	55	85
	AFRP	Expancel sanded	14.9–19.9	5.3	2.8–3.7	91	0.5	55	110
	AFRP	Expancel	14.9–19.9	5.3	2.8–3.7	91	0.5	55	203
[24,38]	AFRP	Smooth braided	64	12.0	5.3	68–76	0.48	34	450
	AFRP	Sanded	63.3	12.0	5.3	68	0.48	39	225
[36,37]	GFRP	Ribbed	55	12.0	4.6	60	0.36	31	221
[50]	CFRP	Spirally indented, sanded, cross wound	46	8.0	5.8	130–144	0.6	45	300
	CFRP	Helical plain	46	8.0	5.8	147	0.6	45	400
	AFRP	Spirally indented	46	8.0	5.8	54	0.6	45	500
	GFRP	Spirally indented	46	8.0	5.8	47	0.6	45	500

c—concrete cover, Ø—reinforcement diameter, E_p_—modulus of elasticity of reinforcement, f_pi_—initial prestress of reinforcement, f_pu_—tensile strength of reinforcement, f_ci_—concrete compressive strength at release, L_t_—transfer length.

**Table 7:** Summary of transfer length results on the influence of modulus of elasticity of reinforcement.

Reference	FRP Type	FRP Surface	c, mm	Ø, mm	c/Ø	f_pi_/f_pu_	f_ci_, MPa	E_p_, GPa	L_t._, mm
[33]	CFRP	Spirally indented	61	8.0	7.6	0.4	26	172	825
	CFRP	Spirally indented	61	8.0	7.6	0.4	63	172	355
	AFRP	Sanded	61	7.8	7.8	0.37	27	54	220
	AFRP	Sanded	61	7.8	7.8	0.37	64	54	170
[47]	CFRP	Spirally indented	64.7	7.9	8.2	0.58–0.59	28–29	150	432
	CFCC	Helical plain	46.9–64.5	8.3	5.6–7.8	0.53–0.59	28–30	137	432
	AFRP	Rough	47.3–64.9	7.9	6.4–8.8	0.51–0.57	30	69	327
[53,54]	CFRP	Spirally indented	40.6	7.9	5.1	0.61	40	171	422
	AFRP	Rough	40.6	7.9	5.1	0.62	43	45	368
[51,52]	GFRP	Helical plain	46.3	9.5	4.9	0.43	32	69	254
	GFRP	Helical plain	46.3	9.5	4.9	0.46	40	69	267
[36,37]	GFRP	Ribbed	55	12.0	4.6	0.36	31	60	221
	GFRP	Ribbed	55	16.0	3.4	0.41	31	60	278

c—concrete cover, Ø—reinforcement diameter, E_p_—modulus of elasticity of reinforcement, f_pi_—initial prestress of reinforcement, f_pu_—tensile strength of reinforcement, f_ci_—concrete compressive strength at release, L_t_—transfer length.

**Table 8:** Summary of the theoretical models for the transfer length of FRP reinforcement.

Reference	Equation	Equation No.	Notes
[10,12,31,32]	$L_{t}=\frac{f_{pi}\cdot Ø}{\alpha_{t}\cdot f_{ci}^{\frac{2}{3}}}$	(1)	L_t_—is the transfer length, f_pi_—is the initial prestress level, Ø—is the reinforcement diameter, f_ci_—is the concrete compressive strength at the time of transfer, α_t_—is a material dependent coefficient.
[33]	$L_{t}=\frac{\kappa \cdot Ø}{\sqrt{f_{ci}}}$	(2)	κ—is a factor equal to 480 in N·mm units (for the CFRP Leadline bar with a spirally indented surface), Ø—is the reinforcement diameter, f_ci_—is the concrete compressive strength at the time of transfer.
[30]	$L_{t}=\frac{f_{pe}\cdot A_{p}}{C_{T}\cdot \sqrt{f_{ci}}}$	(3)	A_p_—cross-sectional area of prestressed reinforcement, f_pe_—effective prestressing stress in the CFCC strand, C_T_—constant is equal to 80 for CFCC strands, f_ci_—is the concrete compressive strength at the time of transfer.
[70]	$L_{t}=\frac{2.5\cdot A_{p}\cdot f_{pi}}{f_{ci}^{\frac{2}{3}}}$	(4)	A_p_—cross-sectional area of prestressed reinforcement, f_pi_—is the initial prestress level, f_ci_—is the concrete compressive strength at the time of transfer.
[64]	$L_{t}=\frac{f_{pi}\cdot Ø}{20.7}$	(5)	f_pi_—is the initial prestress level, Ø—is the reinforcement diameter.
[63]	$L_{t}=\frac{f_{pi}\cdot \emptyset }{20.7}\cdot \sqrt{\frac{20.7}{f_{ci}}}$	(6)	f_pi_—is the initial prestress level, Ø—is the reinforcement diameter, f_ci_—is the concrete compressive strength at the time of transfer.

**Table 9:** Summary of material dependent coefficient α_t_.

Reference	Reinforcement Type	α_t_
[10,12,31,32]	CFRP Leadline bar	1.9
	CFCC strands	4.8
[39]	CFRP Leadline bar	1.95
	CFCC strands	2.12
[36,37]	GFRP bars	2.6
[43,44]	CFRP bars (SCC concrete)	$\alpha_{t}=2.84-\frac{f_{pi}}{800}$

**Table 10:** Summary of initial parameters of the transfer length database.

FRP Type	c, mm	Ø, mm	c/Ø	A_p_, mm^2^	E_p_, GPa	f_pu_, MPa	f_pi_, MPa	f_pi_/f_pu_	f_ci_, MPa
GFRP	25–55	9.5–16	1,6–4.9	45.3–201	55–69.2	1100–1941	313–903	0.26–0.47	29–71
CFCC	38.2–76.2	8.3–15.2	2.9–7.8	54.2–115.6	137–155	1725–2348	561–1526	0.3–0.81	22–56.3
CFRP	19.8–100	5.3–12.7	2.5–10	22.1–126.7	124.1–172	1526–2845	600–1930	0.26–0.86	26–101.2
AFRP	12.4–66	5.3–16	2.3–8.8	11.1–180	44.8–127.5	1134–3000	349–1486	0.23–0.82	27–81.5
BFRP	46–58	8–12	4.8–5.8	50.2–113.1	36–44	920–1126	369–442	0.31–0.48	27–52.4

**Table 11:** Results for the coefficient α_t_.

α_t_	GFRP	CFCC		CFRP	AFRP			BFRP
		Gradual Release	Sudden Release		All	Smooth Braided	Sanded and Rough	
Mean	2.58	4.99	2.42	1.92	2.90	1.53	3.99	2.1
Standard deviation	0.33	0.62	0.67	0.48	1.80	0.55	1.70	1.7
Coefficient of variation, %	12.8	12.5	27.7	24.8	62.1	35.8	42.7	79.2

**Table 12:** Summary of the statistical results for L_t.teor_/L_t.exp_.

Parameters	GFRP	CFCC					CFRP			AFRP		
		Gradual			Sudden					All	Smooth Braided	Sanded and Rough
	α_t_ = 2.6	α_t_ = 5.0	α_t_ = 4.8	α_t_ = 2.12	α_t_ = 2.4	α_t_ = 2.12	α_t_ = 1.90	α_t_ = 1.92	α_t_ = 1.95	α_t_ = 2.9	α_t_ = 1.5	α_t_ = 4.0
M	1.00	1.00	1.04	2.35	1.01	1.14	1.01	1.00	0.98	1.00	1.02	1.00
STD	0.13	0.12	0.13	0.29	0.28	0.32	0.25	0.25	0.24	0.62	0.36	0.43
COV, %	12.6	12.5	12.5	12.5	27.7	27.7	24.8	24.8	24.8	62.1	35.8	42.7
N_total_	26	41	41	41	21	21	73	73	73	70	31	39
OV	17	21	28	41	8	11	40	40	39	29	14	20
UV	9	20	13	0	13	10	33	33	34	41	17	19
M+STD	1.13	1.12	1.17	2.65	1.29	1.46	1.26	1.25	1.23	1.62	1.38	1.42
M-STD	0.87	0.88	0.91	2.06	0.73	0.83	0.76	0.75	0.74	0.38	0.65	0.57
>M+STD	3	6	6	6	5	5	8	8	8	15	3	10
<M-STD	2	8	8	8	2	2	13	13	13	9	9	10
Inbound	21	27	27	27	14	14	52	52	52	46	19	19
Inbound, %	80.8	65.9	65.9	65.9	66.7	66.7	71.2	71.2	71.2	65.7	61.3	48.7

M—mean L_t.teor_/L_t.exp_, STD—standard deviation, COV—coefficient of variation, N_total_—the total amount of specimens, UV—underestimated values, OV—overestimated values, M+STD—upper bound, M-STD—lower bound.

## Data Availability

Not applicable.

## Appendix A

**Table A1:** Geometrical, mechanical properties of the material and transfer lengths of specimens with pretensioned GFRP reinforcement.

Reference	Specimen No.	FRP Type	FRP Surface	Specimen Type	Release Type	Shear Reinforcement	Specimen Dimensions (b × h × l, mm)	c, mm	Ø, mm	A_p_, mm^2^	E_p_, GPa	f_pu_, MPa	f_pi_, MPa	f_pi_/f_pu_	f_ci_, MPa	L_t_, mm
[36,37]	N40-16-1	GFRP Bar	Ribbed	Beam	Gradual	Yes	150 × 255 × 3600	55.0	16.0	201.0	60	1200	490	0.41	31.0	287.5
	N40-16-2			Beam	Gradual	Yes	150 × 255 × 3600	55.0	16.0	201.0	60	1200	490	0.41	31.0	285.0
	N40-16-3			Beam	Gradual	Yes	150 × 255 × 3600	55.0	16.0	201.0	60	1200	490	0.41	31.0	270.0
	N40-16-4			Beam	Gradual	Yes	150 × 255 × 3600	55.0	16.0	201.0	60	1200	490	0.41	31.0	270.0
	N40-12-1			Beam	Gradual	Yes	150 × 255 × 3600	55.0	12.0	113.0	60	1350	490	0.36	31.0	225.0
	N40-12-2			Beam	Gradual	Yes	150 × 255 × 3600	55.0	12.0	113.0	60	1350	490	0.36	31.0	220.0
	N40-12-3			Beam	Gradual	Yes	150 × 255 × 3600	55.0	12.0	113.0	60	1350	490	0.36	31.0	210.0
	N40-12-4			Beam	Gradual	Yes	150 × 255 × 3600	55.0	12.0	113.0	60	1350	490	0.36	31.0	227.5
	H40-16-1			Beam	Gradual	Yes	150 × 255 × 3600	55.0	16.0	201.0	60	1200	500	0.42	71.0	177.5
	H40-16-2			Beam	Gradual	Yes	150 × 255 × 3600	55.0	16.0	201.0	60	1200	500	0.42	71.0	200.0
	H40-16-3			Beam	Gradual	Yes	150 × 255 × 3600	55.0	16.0	201.0	60	1200	500	0.42	71.0	187.5
	H40-16-4			Beam	Gradual	Yes	150 × 255 × 3600	55.0	16.0	201.0	60	1200	500	0.42	71.0	187.5
	H25-16-1			Beam	Gradual	Yes	150 × 255 × 3600	55.0	16.0	201.0	60	1200	313	0.26	29.0	215.0
	H25-16-2			Beam	Gradual	Yes	150 × 255 × 3600	55.0	16.0	201.0	60	1200	313	0.26	29.0	207.5
	H25-16-3			Beam	Gradual	Yes	150 × 255 × 3600	55.0	16.0	201.0	60	1200	313	0.26	29.0	212.5
	H25-16-4			Beam	Gradual	Yes	150 × 255 × 3600	55.0	16.0	201.0	60	1200	313	0.26	29.0	210.0
[71]	SC-12-1.5	GFRP Bar	Sanded	Beam	Gradual	Yes	200 × 235 × 2000	25.0	12.0	138.0	55	1434	536	0.37	46.3	300.0
	R-12-1.5		Ribbed	Beam	Gradual	Yes	201 × 235 × 2000	25.0	12.0	113.0	55	1350	520	0.39	47.2	300.0
	SC-16-1.5		Sanded	Beam	Gradual	Yes	202 × 235 × 2000	25.0	16.0	196.0	61	1180	452	0.38	46.3	200.0
	SC-16-3		Sanded	Beam	Gradual	Yes	203 × 240 × 2000	45.0	16.0	196.0	61	1180	453	0.38	47.8	200.0
	R-16-1.5		Ribbed	Beam	Gradual	Yes	204 × 235 × 2000	25.0	16.0	201.0	60	1100	426	0.39	47.2	200.0
[51]	FG-W4	GFRP 7-wire Strand	Helical plain	Prism	Sudden	No	152 × 102 × 2590	46.3	9.5	45.3	69	1941	899	0.46	40.1	279.4
	FG-M4			Prism	Sudden	No	153 × 102 × 2590	46.3	9.5	45.3	69	1941	899	0.46	40.1	254.0
	FG-E4			Prism	Sudden	No	154 × 102 × 2590	46.3	9.5	45.3	69	1941	899	0.46	40.1	279.4
	FG-E5			Prism	Sudden	No	155 × 102 × 2590	46.3	9.5	45.3	69	1941	904	0.47	40.1	254.0
	FG-M7			Prism	Sudden	No	156 × 102 × 2590	46.3	9.5	45.3	69	1941	825	0.43	32.6	254.0

**Table A2:** Geometrical, mechanical properties of the material and transfer lengths of specimens with pretensioned CFCC reinforcement.

Reference	Specimen No	FRP Type	FRP Surface	Specimen Type	Release Type	Shear Reinforcement	Specimen Dimensions (b × h × l, mm)	c, mm	Ø, mm	A_p_, mm^2^	E_p_, GPa	f_pu_, MPa	f_pi_, MPa	f_pi_/f_pu_	f_ci_, MPa	L_t_, mm
[29,30,58,66]	BBC-I-1	CFCC 7-wire Strand	Helical plain	Box beam	Sudden	Yes	230 × 970 × 6100	50.0	12.7	77.4	142	1869	565	0.30	42.6	266.7
	BBC-I-2			Box beam	Sudden	Yes	231 × 970 × 6100	50.0	12.7	77.4	142	1869	561	0.30	42.6	292.1
	BBC-II-1			Box beam	Sudden	Yes	232 × 970 × 6100	50.0	12.7	77.4	142	1869	564	0.30	43.3	330.2
	BBC-II-2			Box beam	Sudden	Yes	233 × 970 × 6100	50.0	12.7	77.4	142	1869	587	0.31	43.3	304.8
	BBC-III-1			Box beam	Sudden	Yes	234 × 970 × 6100	50.0	12.7	77.4	142	1869	596	0.32	44.0	317.5
	BBC-III-2			Box beam	Sudden	Yes	235 × 970 × 6100	50.0	12.7	77.4	142	1869	597	0.32	44.0	368.3
[29,30]	A type-B6	CFCC 7-wire Strand	Helical plain	T Beam	Gradual	Yes	T	50.0	12.5	76.0	137	1870	933	0.50	56.3	126.5
	A type-B7			T Beam	Gradual	Yes	T	50.0	12.5	76.0	137	1870	955	0.51	56.3	148.5
	A type-B8			T Beam	Gradual	Yes	T	50.0	12.5	76.0	137	1870	895	0.48	41.4	149.5
	B type-D10			T Beam	Gradual	Yes	T	75.0	12.5	76.0	137	1870	909	0.49	41.4	167.5
	A type-C9			T Beam	Gradual	Yes	T	50.0	12.5	76.0	137	1870	1024	0.55	50.0	147.5
	D type-BT7			Beam	Gradual	Yes	100 × 250	50.0	12.5	76.0	137	1870	1403	0.75	30.0	400.0
	D type-BT8			Beam	Gradual	Yes	100 × 250	50.0	12.5	76.0	137	1870	1403	0.75	30.0	375.0
	D type-BT9			Beam	Gradual	Yes	100 × 250	50.0	12.5	76.0	137	1870	1403	0.75	30.0	350.0
	D type-BT10			Beam	Gradual	Yes	100 × 250	50.0	12.5	76.0	137	1870	1403	0.75	30.0	350.0
	A type-A1			T Beam	Gradual	Yes	T	50.0	15.2	113.6	137	1750	838	0.48	35.8	-
	A type-A2			T Beam	Gradual	Yes	T	50.0	15.2	113.6	137	1750	851	0.49	35.8	-
	A type-A3			T Beam	Gradual	Yes	T	50.0	15.2	113.6	137	1750	851	0.49	40.2	223.0
	B type-D5			T Beam	Gradual	Yes	T	75.0	15.2	113.6	137	1750	877	0.50	40.2	200.5
	C type-BT1			Beam	Gradual	Yes	120 × 300	60.0	15.2	113.6	137	1750	1050	0.60	30.0	370.0
	C type-BT2			Beam	Gradual	Yes	120 × 300	60.0	15.2	113.6	137	1750	1050	0.60	30.0	370.0
	A type-C4			T Beam	Gradual	Yes	T	50.0	15.2	113.6	137	1750	1212	0.69	45.1	232.0
	C type-BT3			Beam	Gradual	Yes	120 × 300	60.0	15.2	113.6	137	1750	1313	0.75	30.0	400.0
	C type-BT4			Beam	Gradual	Yes	120 × 300	60.0	15.2	113.6	137	1750	1313	0.75	30.0	387.5
	C type-BT5			Beam	Gradual	Yes	120 × 300	60.0	15.2	113.6	137	1750	1313	0.75	30.0	412.5
	C type-BT6			Beam	Gradual	Yes	120 × 300	60.0	15.2	113.6	137	1750	1313	0.75	30.0	412.5
[31,32]	BT11	CFCC 7-wire Strand	Helical plain	A1 Beam	Gradual	Yes	80 × 250 × 1380	45.0	10.5	55.7	140	1725	1400	0.81	29.0	305.0
	BT12			A2 Beam	Gradual	Yes	80 × 250 × 1381	45.0	10.5	55.7	140	1725	1400	0.81	29.0	312.5
	BT13			A1 Beam	Gradual	Yes	80 × 250 × 1382	45.0	10.5	55.7	140	1725	1400	0.81	29.0	312.5
	BT14			A2 Beam	Gradual	Yes	80 × 250 × 1383	45.0	10.5	55.7	140	1725	1400	0.81	29.0	312.5
	BT7			A1 Beam	Gradual	Yes	100 × 250 × 1380	50.0	12.5	76.0	141	1868	1408	0.75	28.0	400.0
	BT8			B Beam	Gradual	Yes	100 × 250 × 1381	50.0	12.5	76.0	141	1868	1408	0.75	28.0	375.0
	BT9			B Beam	Gradual	Yes	100 × 250 × 1382	50.0	12.5	76.0	141	1868	1408	0.75	28.0	360.0
	BT10			B Beam	Gradual	Yes	100 × 250 × 1383	50.0	12.5	76.0	141	1868	1408	0.75	28.0	360.0
	BT15			C1, C2 Beam	Gradual	Yes	100 × 250 × 2750	50.0	12.5	76.0	141	1868	1125	0.60	30.0	300.0
	BT16			C1, C2 Beam	Gradual	Yes	100 × 250 × 2751	50.0	12.5	76.0	141	1868	1125	0.60	30.0	295.0
	BT19			C5 Beam	Gradual	No	175 × 250 × 2750	50.0	12.5	76.0	141	1868	1355	0.73	23.0	500.0
	BT20			C5 Beam	Gradual	No	175 × 250 × 2750	50.0	12.5	76.0	141	1868	1355	0.73	23.0	450.0
	BT1			A1 Beam	Gradual	Yes	120 × 300 × 1720	60.0	15.2	113.6	138	1750	1074	0.61	35.0	365.0
	BT2			A1 Beam	Gradual	Yes	120 × 300 × 1720	60.0	15.2	113.6	138	1750	1074	0.61	35.0	355.0
	BT3			A1 Beam	Gradual	Yes	120 × 300 × 1720	60.0	15.2	113.6	138	1750	1312	0.75	31.0	392.5
	BT4			B Beam	Gradual	Yes	120 × 300 × 1721	60.0	15.2	113.6	138	1750	1312	0.75	31.0	387.5
	BT5			A2 Beam	Gradual	Yes	120 × 300 × 1722	60.0	15.2	113.6	138	1750	1312	0.75	31.0	400.0
	BT6			A3 Beam	Gradual	Yes	120 × 300 × 1723	60.0	15.2	113.6	138	1750	1312	0.75	31.0	400.0
	BT17			C3, C4 Beam	Gradual	No	200 × 300 × 3500	60.0	15.2	113.6	138	1750	1294	0.74	22.0	650.0
	BT18			C5 Beam	Gradual	No	200 × 300 × 3500	60.0	15.2	113.6	138	1750	1294	0.74	22.0	600.0
	BT21			C5 Beam	Gradual	No	175 × 300 × 3500	60.0	15.2	113.6	138	1750	1083	0.62	24.0	510.0
	BT22			C3, C4 Beam	Gradual	No	175 × 300 × 3500	60.0	15.2	113.6	138	1750	1083	0.62	24.0	490.0
[59,60,61]	5S	CFCC 7-wire Strand	Helical plain	Pile	Sudden	Yes	610 × 610 × 12200	43.4	15.2	115.0	155	2348	1526	0.65	37.0	647.7
	3N			Pile	Sudden	Yes	610 × 610	76.2	15.2	115.6	155	2336	1518	0.65	37.0	736.6
	3S			Pile	Sudden	Yes	611 × 610	76.2	15.2	115.6	155	2336	1518	0.65	37.0	546.1
	4N			Pile	Sudden	Yes	612 × 610	76.2	15.2	115.6	155	2336	1518	0.65	37.0	647.7
	4S			Pile	Sudden	Yes	613 × 610	76.2	15.2	115.6	155	2336	1518	0.65	37.0	558.8
	5N			Pile	Sudden	Yes	614 × 610	76.2	15.2	115.6	155	2336	1518	0.65	37.0	711.2
	5S			Pile	Sudden	Yes	615 × 610	76.2	15.2	115.6	155	2336	1518	0.65	37.0	622.3
[47]	CT-2	CFCC 7-wire Strand	Helical plain	Prism	Gradual	No	106 × 102 × 3000	46.9	8.3	54.2	137	2220	1305	0.59	28.1	279.4
	CT-1 (J)			Beam	Gradual	No	143 × 203 × 6000	64.5	8.3	54.2	137	2220	1174	0.53	30.6	457.2
	CT-1 (D)			Beam	Gradual	No	144 × 203 × 6000	64.5	8.3	54.2	137	2220	1174	0.53	30.6	558.8
	2CT-1 (J)			Beam	Gradual	No	145 × 203 × 6000	64.5	8.3	54.2	137	2220	681	0.31	30.8	558.8
	2CT-1 (D)			Beam	Gradual	No	146 × 203 × 6000	64.5	8.3	54.2	137	2220	681	0.31	30.8	330.2
[39]	CDT2-1-WA-A1	CFCC 7-wire Strand	Helical plain	Girder	Sudden	Yes	Double T	38.2	12.5	76.0	138	1868	925	0.50	48.0	501.5
	CDT2-1-WA-A2			Girder	Sudden	Yes	Double T	38.2	12.5	76.0	138	1869	901	0.48	48.0	-
	CDT2-1-WA-A3			Girder	Sudden	Yes	Double T	38.2	12.5	76.0	138	1870	878	0.47	48.0	-
	CDT2-1-WA-B1			Girder	Sudden	Yes	Double T	38.2	12.5	76.0	138	1871	954	0.51	48.0	419.0
	CDT2-1-WA-B2			Girder	Sudden	Yes	Double T	38.2	12.5	76.0	138	1872	954	0.51	48.0	-
	CDT2-1-WA-B3			Girder	Sudden	Yes	Double T	38.2	12.5	76.0	138	1873	913	0.49	48.0	-
	CDT2-2-WA-A1			Girder	Sudden	Yes	Double T	38.2	12.5	76.0	138	1874	954	0.51	48.0	482.5
	CDT2-2-WA-A2			Girder	Sudden	Yes	Double T	38.2	12.5	76.0	138	1875	959	0.51	48.0	-
	CDT2-2-WA-A3			Girder	Sudden	Yes	Double T	38.2	12.5	76.0	138	1876	942	0.50	48.0	-
	CDT2-2-WA-B1			Girder	Sudden	Yes	Double T	38.2	12.5	76.0	138	1877	913	0.49	48.0	468.5
	CDT2-2-WA-B2			Girder	Sudden	Yes	Double T	38.2	12.5	76.0	138	1878	924	0.49	48.0	-
	CDT2-2-WA-B3			Girder	Sudden	Yes	Double T	38.2	12.5	76.0	138	1879	977	0.52	48.0	-
	CDT2-3-WA-A1			Girder	Sudden	Yes	Double T	38.2	12.5	76.0	138	1880	1018	0.54	48.0	520.5
	CDT2-3-WA-A2			Girder	Sudden	Yes	Double T	38.2	12.5	76.0	138	1881	966	0.51	48.0	-
	CDT2-3-WA-A3			Girder	Sudden	Yes	Double T	38.2	12.5	76.0	138	1882	983	0.52	48.0	-
	CDT2-3-WA-B1			Girder	Sudden	Yes	Double T	38.2	12.5	76.0	138	1883	972	0.52	48.0	412.5
	CDT2-3-WA-B2			Girder	Sudden	Yes	Double T	38.2	12.5	76.0	138	1884	1001	0.53	48.0	-
	CDT2-3-WA-B3			Girder	Sudden	Yes	Double T	38.2	12.5	76.0	138	1885	1036	0.55	48.0	-
	CDT2-4-WA-A1			Girder	Sudden	Yes	Double T	38.2	12.5	76.0	138	1886	983	0.52	48.0	443.0
	CDT2-4-WA-A2			Girder	Sudden	Yes	Double T	38.2	12.5	76.0	138	1887	1095	0.58	48.0	-
	CDT2-4-WA-A3			Girder	Sudden	Yes	Double T	38.2	12.5	76.0	138	1888	995	0.53	48.0	-
	CDT2-4-WA-B1			Girder	Sudden	Yes	Double T	38.2	12.5	76.0	138	1889	1059	0.56	48.0	400.0
	CDT2-4-WA-B2			Girder	Sudden	Yes	Double T	38.2	12.5	76.0	138	1890	1024	0.54	48.0	-
	CDT2-4-WA-B3			Girder	Sudden	Yes	Double T	38.2	12.5	76.0	138	1891	1240	0.66	48.0	266.7

**Table A3:** Geometrical, mechanical properties of the material and transfer lengths of specimens with pretensioned CFRP reinforcement.

Reference	Specimen No	FRP Type	FRP Surface	Specimen Type	Release Type	Shear Reinforcement	Specimen Dimensions (b × h × l, mm)	c, mm	Ø, mm	A_p_, mm^2^	E_p_, GPa	f_pu_, MPa	f_pi_, MPa	f_pi_/f_pu_	f_ci_, MPa	L_t_, mm
[53,54]	CL	CFRP Leadline	Spirally Indented	Beam	-	No	89 × 178 × 3000	40.6	7.9	47.1	171	2255	1365	0.61	40.0	421.6
	CS	CFRP Custom		Beam	-	No	89 × 178 × 3000	40.6	7.9	50.3	161	1834	1172	0.64	35.5	408.9
[58,65]	BBD-I_BP-1	CFRP-DCITendon	Spirally Indented	Box beam	Sudden	Yes	-	50.0	9.5	70.9	131	1930	635	0.33	33.8	241.5
	BBD-I_BP-2			Box beam	Sudden	Yes	-	50.0	9.5	70.9	131	1930	635	0.33	33.8	279.5
	BBD-II_B0-1			Box beam	Sudden	Yes	-	50.0	9.5	70.9	131	1930	635	0.34	34.8	266.5
	BBD-II_B0-2			Box beam	Sudden	Yes	-	50.0	9.5	70.9	131	1930	635	0.33	34.8	279.5
	BBD-III_BN-1			Box beam	Sudden	Yes	-	50.0	9.5	70.9	131	1930	635	0.33	35.2	304.5
	BBD-III_BN-2			Box beam	Sudden	Yes	-	50.0	9.5	70.9	131	1930	635	0.32	35.2	279.0
[72]	-	CFRP Leadline	Spirally Indented	Double T Beam	Sudden	Yes	-	100.0	10.0	71.6	147	2262	1180	0.52	45.9	381.0
[45]	B1-4-65	CFRP Leadline	Spirally indented sanded	Beam	Gradual	Yes	139.7 × 247.7 × 4000	31.8	12.7	126.5	124	2275	1345	0.59	45.4	607.1
	B2-4-55			Beam	Gradual	Yes	139.7 × 247.7 × 4001	31.8	12.7	126.5	124	2275	1138	0.50	47.9	533.4
	B3-4-60			Beam	Gradual	Yes	139.7 × 247.7 × 4002	31.8	12.7	126.5	124	2275	1241	0.55	46.8	495.3
	B4-4-60			Beam	Gradual	Yes	139.7 × 247.7 × 4003	31.8	12.7	126.5	124	2275	1282	0.56	50.7	342.9
[31,32]	BL1	CFRP Leadline	Spirally Indented	Beam	Gradual	Yes	80 × 200 × 1700	35.0	8.0	46.1	147	1970	1193	0.61	34.0	452.5
	BL2			Beam	Gradual	Yes	80 × 200 × 1701	35.0	8.0	46.1	147	1970	1193	0.61	34.0	462.5
	BL3			Beam	Gradual	Yes	80 × 200 × 1702	35.0	8.0	46.1	147	1970	1410	0.72	35.0	480.0
	BL4			Beam	Gradual	Yes	80 × 200 × 1703	35.0	8.0	46.1	147	1970	1410	0.72	35.0	480.0
	BL5			Beam	Gradual	Yes	80 × 250 × 2500	35.0	8.0	46.1	147	1970	1497	0.76	28.0	620.0
	BL6			Beam	Gradual	Yes	80 × 250 × 1700	35.0	8.0	46.1	147	1970	1497	0.76	28.0	625.0
	BL7			Beam	Gradual	Yes	80 × 250 × 2100	35.0	8.0	46.1	147	1970	1497	0.76	28.0	610.0
	BL8			Beam	Gradual	Yes	80 × 250 × 2100	35.0	8.0	46.1	147	1970	1497	0.76	28.0	625.0
	BL9			Beam	Gradual	No	150 × 250 × 4000	36.0	8.0	46.1	147	1970	1258	0.64	26.0	700.0
	BL10			Beam	Gradual	No	150 × 250 × 4000	36.0	8.0	46.1	147	1970	1258	0.64	26.0	700.0
	BL11			Beam	Gradual	No	150 × 250 × 3900	36.0	8.0	46.1	147	1970	1518	0.77	42.0	600.0
	BL12			Beam	Gradual	No	150 × 250 × 3900	36.0	8.0	46.1	147	1970	1518	0.77	42.0	500.0
	PL1			Prism	Gradual	No	70 × 95 × 1884	35.0	8.0	46.1	147	1970	1193	0.61	34.0	490.0
	PL2			Prism	Gradual	No	70 × 95 × 1884	35.0	8.0	46.1	147	1970	1193	0.61	34.0	480.0
	PL3			Prism	Gradual	No	80 × 80 × 1884	40.0	8.0	46.1	147	1970	1410	0.72	31.0	540.0
	PL4			Prism	Gradual	No	80 × 80 × 1884	40.0	8.0	46.1	147	1970	1410	0.72	31.0	525.0
[33]	C40-80-L-C	CFRP Leadline	Spirally Indented	Beam	Gradual	Yes	150 × 300 × 6000	61.0	8.0	50.2	172	2845	789	0.28	26.0	750.0
	C40-120-L-C			Beam	Gradual	Yes	151 × 300 × 6000	61.0	8.0	50.2	172	2845	1213	0.43	26.0	825.0
	C40-80-S-S			Beam	Gradual	Yes	152 × 300 × 6000	61.0	8.0	50.2	172	2845	834	0.29	35.0	480.0
	C40-80-L-U			Beam	Gradual	Yes	153 × 300 × 6000	61.0	8.0	50.2	172	2845	819	0.29	35.0	530.0
	C40-120-L-U			Beam	Gradual	Yes	154 × 300 × 6000	61.0	8.0	50.2	172	2845	1225	0.43	37.0	675.0
	C80-120-L-U			Beam	Gradual	Yes	156 × 300 × 6000	61.0	8.0	50.2	172	2845	1217	0.43	55.0	655.0
	C80-120-S-S			Beam	Gradual	Yes	158 × 300 × 6000	61.0	8.0	50.2	172	2845	1141	0.40	63.0	355.0
	C80-80-L-C			Beam	Gradual	Yes	159 × 300 × 6000	61.0	8.0	50.2	172	2845	745	0.26	63.0	305.0
[40,41,42]	1	CFRP Leadline	Spirally Indented	T Beam	Gradual	Yes	T	46.1	7.8	47.3	147	1970	980	0.50	47.0	360.0
	2			T Beam	Gradual	Yes	T	46.1	7.8	47.3	147	1970	1380	0.70	50.0	500.0
[47]	CL-2	CFRP Leadline	Spirally Indented	Prism	Gradual	No	105 × 102 × 3000	47.1	7.9	46.5	150	1979	1100	0.56	28.1	939.8
	CL-1 (J)			Beam	Gradual	No	137 × 203 × 6000	64.7	7.9	46.5	150	1979	1302	0.66	28.3	-
	CL-1 (D)			Beam	Gradual	No	138 × 203 × 6000	64.7	7.9	46.5	150	1979	1302	0.66	28.3	-
	2CL-1 (J)			Beam	Gradual	No	139 × 203 × 6000	64.7	7.9	46.5	150	1979	1169	0.59	29.0	304.8
	2CL-1 (D)			Beam	Gradual	No	140 × 203 × 6000	64.7	7.9	46.5	150	1979	1169	0.59	29.0	381.0
	CL-3 (J)			Beam	Gradual	No	141 × 203 × 6000	64.7	7.9	46.5	150	1979	1139	0.58	28.3	431.8
	CL-3 (D)			Beam	Gradual	No	142 × 203 × 6000	64.7	7.9	46.5	150	1979	1139	0.58	28.3	609.6
[46]	T1	CFRP Leadline	Spirally Indented	T Beam	Gradual	Yes	T	46.1	7.9	46.1	150	2300	1128	0.49	30.0	650.0
	T2			T Beam	Gradual	Yes	T	46.1	7.9	46.1	150	2300	1578	0.69	30.0	725.0
	T3-1			T Beam	Gradual	Yes	T	46.1	7.9	46.1	150	2300	1150	0.50	30.0	675.0
	T3-2			T Beam	Gradual	Yes	T	46.1	7.9	46.1	150	2300	1610	0.70	30.0	675.0
	R1-R4			Beam	Gradual	Yes	150 × 300	46.1	7.9	46.1	150	2300	1356	0.59	30.0	625.0
[39]	CDT1-1-WA-A1	CFRP Leadline	Spirally Indented	Girder	Gradual	Yes	Double T	40.5	7.9	46.1	147	2256	1740	0.77	48.0	557.5
	CDT1-1-WA-A2			Girder	Gradual	Yes	Double T	40.5	7.9	46.1	147	2256	1830	0.81	48.0	-
	CDT1-1-WA-A3			Girder	Gradual	Yes	Double T	40.5	7.9	46.1	147	2256	1300	0.58	48.0	-
	CDT1-1-WA-B1			Girder	Gradual	Yes	Double T	40.5	7.9	46.1	147	2256	1540	0.68	48.0	507.5
	CDT1-1-WA-B2			Girder	Gradual	Yes	Double T	40.5	7.9	46.1	147	2256	1540	0.68	48.0	-
	CDT1-1-WA-B3			Girder	Gradual	Yes	Double T	40.5	7.9	46.1	147	2256	1360	0.60	48.0	-
	CDT1-2-WA-A1			Girder	Sudden	Yes	Double T	40.5	7.9	46.1	147	2256	1640	0.73	48.0	470.0
	CDT1-2-WA-A2			Girder	Sudden	Yes	Double T	40.5	7.9	46.1	147	2256	1740	0.77	48.0	-
	CDT1-2-WA-A3			Girder	Sudden	Yes	Double T	40.5	7.9	46.1	147	2256	1120	0.50	48.0	-
	CDT1-2-WA-B1			Girder	Sudden	Yes	Double T	40.5	7.9	46.1	147	2256	1540	0.68	48.0	-
	CDT1-2-WA-B2			Girder	Sudden	Yes	Double T	40.5	7.9	46.1	147	2256	1640	0.73	48.0	-
	CDT1-2-WA-B3			Girder	Sudden	Yes	Double T	40.5	7.9	46.1	147	2256	1120	0.50	48.0	-
	CDT1-3-WA-A1			Girder	Sudden	Yes	Double T	40.5	7.9	46.1	147	2256	1640	0.73	48.0	502.5
	CDT1-3-WA-A2			Girder	Sudden	Yes	Double T	40.5	7.9	46.1	147	2256	1640	0.73	48.0	-
	CDT1-3-WA-A3			Girder	Sudden	Yes	Double T	40.5	7.9	46.1	147	2256	1050	0.47	48.0	-
	CDT1-3-WA-B1			Girder	Sudden	Yes	Double T	40.5	7.9	46.1	147	2256	1930	0.86	48.0	415.0
	CDT1-3-WA-B2			Girder	Sudden	Yes	Double T	40.5	7.9	46.1	147	2256	1540	0.68	48.0	-
	CDT1-3-WA-B3			Girder	Sudden	Yes	Double T	40.5	7.9	46.1	147	2256	930	0.41	48.0	-
	CDT1-4-WA-A1			Girder	Sudden	Yes	Double T	40.5	7.9	46.1	147	2256	1640	0.73	48.0	487.5
	CDT1-4-WA-A2			Girder	Sudden	Yes	Double T	40.5	7.9	46.1	147	2256	1640	0.73	48.0	-
	CDT1-4-WA-A3			Girder	Sudden	Yes	Double T	40.5	7.9	46.1	147	2256	1240	0.55	48.0	-
	CDT1-4-WA-B1			Girder	Sudden	Yes	Double T	40.5	7.9	46.1	147	2256	1740	0.77	48.0	502.5
	CDT1-4-WA-B2			Girder	Sudden	Yes	Double T	40.5	7.9	46.1	147	2256	1350	0.60	48.0	-
	CDT1-4-WA-B3			Girder	Sudden	Yes	Double T	40.5	7.9	46.1	147	2256	1240	0.55	48.0	-
[73]	1	CFRP Leadline	Spirally Indented	Beam	Gradual	Yes	150 × 300 × 3000	46.0	8.0	50.2	-	2070	1242	0.6	41.0	662.5
	3			Beam	Gradual	Yes	151 × 300 × 3000	46.0	8.0	50.2	-	2070	1242	0.6	41.0	650.0
	4			Beam	Gradual	Yes	152 × 300 × 3000	46.0	8.0	50.2	-	2070	1242	0.6	41.8	625.0
	6			Beam	Gradual	Yes	153 × 300 × 3000	46.0	8.0	50.2	-	2070	1242	0.6	41.0	561.5
[43,44]	I-SCC30-1	CFRP Bar	Sanded	Beam	Gradual	Yes	150 × 250 × 3600	38.1	12.7	126.7	144	1765	613	0.32	30.4	-
	I-SCC30-2			Beam	Gradual	Yes	150 × 250 × 3601	38.1	12.7	126.7	144	1765	601	0.31	30.4	355.0
	I-SCC30-3			Beam	Gradual	Yes	150 × 250 × 3602	38.1	12.7	126.7	144	1765	726	0.37	41.0	-
	I-SCC30-4			Beam	Gradual	Yes	150 × 250 × 3603	38.1	12.7	126.7	144	1765	647	0.35	41.0	-
	II-SCC45-1			Beam	Gradual	Yes	150 × 250 × 3604	38.1	12.7	126.7	144	1765	807	0.43	35.0	-
	II-SCC45-2			Beam	Gradual	Yes	150 × 250 × 3605	38.1	12.7	126.7	144	1765	884	0.46	35.0	505.0
	II-SCC45-3			Beam	Gradual	Yes	150 × 250 × 3606	38.1	12.7	126.7	144	1765	841	0.45	35.0	530.0
	II-SCC45-4			Beam	Gradual	Yes	150 × 250 × 3607	38.1	12.7	126.7	144	1765	805	0.43	35.0	-
	III-SCC60-1			Beam	Gradual	Yes	150 × 250 × 3608	38.1	12.7	126.7	144	1765	980	0.54	30.4	665.0
	III-SCC60-2			Beam	Gradual	Yes	150 × 250 × 3609	38.1	12.7	126.7	144	1765	1097	0.58	30.4	-
	III-SCC60-3			Beam	Gradual	Yes	150 × 250 × 3610	38.1	12.7	126.7	144	1765	1008	0.54	41.0	695.0
	III-SCC60-4			Beam	Gradual	Yes	150 × 250 × 3611	38.1	12.7	126.7	144	1765	1052	0.57	41.0	680.0
	IV-N30-1			Beam	Gradual	Yes	150 × 250 × 3612	38.1	12.7	126.7	144	1765	635	0.33	37.0	300.0
	IV-N60-2			Beam	Gradual	Yes	150 × 250 × 3613	38.1	12.7	126.7	144	1765	1168	0.63	37.0	-
	IV-N60-3			Beam	Gradual	Yes	150 × 250 × 3614	38.1	12.7	126.7	144	1765	1121	0.60	37.0	580.0
	IV-N60-4			Beam	Gradual	Yes	150 × 250 × 3615	38.1	12.7	126.7	144	1765	1153	0.62	37.0	590.0
[74]	Slab 3	CFRP Bar	Sanded	Slab	-	No	200 × 45 × 3360	19.8	5.4	22.9	150	2000	1200	0.6	101.2	160.0
[34])	C1-UHM	CFRP Bar	Sanded	Beam	Gradual	Yes	90 × 157.5 × 3000	22.5	5.3	22.1	509	1526	800	0.52	62.8	158.0
	C1-UTS			Beam	Gradual	Yes	91 × 157.5 × 3000	22.5	5.3	22.1	145	2031	800	0.39	61.8	86.0
	C4-UHM			Beam	Gradual	Yes	92 × 157.5 × 3000	22.5	5.3	22.1	509	1526	800	0.52	32.9	194.0

**Table A4:** Geometrical, mechanical properties of the material and transfer lengths of specimens with pretensioned AFRP reinforcement.

Reference	Specimen No	FRP Type	FRP Surface	Specimen Type	Release Type	Shear Reinforcement	Specimen Dimensions (b × h × l, mm)	c, mm	Ø, mm	A_p_, mm^2^	E_p_, GPa	f_pu_, MPa	f_pi_, MPa	f_pi_/f_pu_	f_ci_, MPa	L_t_, mm
[57]	Beam1	AFRP Tendon	Smooth braided	Beam	Gradual	No	120 × 210	62.0	16.0	180.0	63	1392	698	0.50	29.1	-
	Beam2			Beam	Gradual	No	121 × 210	62.0	16.0	180.0	63	1392	698	0.50	29.1	-
	Beam3			Beam	Gradual	Yes	122 × 210	62.0	16.0	180.0	63	1392	698	0.50	29.1	625.0
	Beam4			Beam	Gradual	Yes	123 × 210	62.0	16.0	180.0	63	1392	698	0.50	29.1	-
	Beam5			Beam	Gradual	Yes	124 × 210	62.0	16.0	180.0	63	1392	698	0.50	29.1	575.0
	Beam6			Beam	Gradual	Yes	125 × 210	62.0	16.0	180.0	63	1392	698	0.50	29.1	-
	Beam7			Beam	Gradual	No	126 × 210	62.0	16.0	180.0	63	1392	698	0.50	29.1	700.0
	Beam8			Beam	Gradual	No	127 × 210	62.0	16.0	180.0	63	1392	698	0.50	29.1	-
[24,38]	A2-1	AFRP Tendon	Smooth braided	Beam	Gradual	Yes	120 × 210 × 4000	66.0	8.0	42.0	76	1639	374	0.23	37.4	300.0
	A2-2(J)			Beam	Gradual	Yes	121 × 210 × 4000	66.0	8.0	42.0	76	1639	374	0.23	37.5	300.0
	A2-2(F)			Beam	Gradual	Yes	122 × 210 × 4000	66.0	8.0	42.0	76	1639	374	0.23	38.4	300.0
	A2-S3(J)			Beam	Gradual	Yes	123 × 210 × 4000	66.0	8.0	42.0	76	1639	374	0.23	38.6	200.0
	A2-S3(F)			Beam	Gradual	Yes	124 × 210 × 4000	66.0	8.0	42.0	76	1639	374	0.23	38.7	200.0
	A2-S4(F)			Beam	Gradual	Yes	125 × 210 × 4000	66.0	8.0	42.0	76	1639	748	0.46	33.1	400.0
	A2-S4(J)			Beam	Gradual	Yes	126 × 210 × 4000	66.0	8.0	42.0	76	1639	748	0.46	33.4	400.0
	B1-1(F)			Beam	Gradual	Yes	127 × 210 × 4000	64.0	12.0	90.0	68	1461	349	0.24	37.8	400.0
	B1-1(J)			Beam	Gradual	Yes	128 × 210 ×4000	64.0	12.0	90.0	68	1461	349	0.24	37.9	400.0
	B1-2(F)			Beam	Gradual	Yes	129 × 210 × 4000	64.0	12.0	90.0	68	1461	349	0.24	35.8	400.0
	B1-2(J)			Beam	Gradual	Yes	130 × 210 × 4000	64.0	12.0	90.0	68	1461	349	0.24	38.6	400.0
	B1-S3(J)			Beam	Gradual	Yes	131 × 210 × 4000	64.0	12.0	90.0	68	1461	349	0.24	37.6	300.0
	B1-S3(F)			Beam	Gradual	Yes	132 × 210 × 4000	64.0	12.0	90.0	68	1461	349	0.24	37.8	300.0
	B1-S6(J)			Beam	Gradual	Yes	133 × 210 × 4000	64.0	12.0	90.0	68	1461	349	0.24	38.0	300.0
	B1-S6(F)			Beam	Gradual	Yes	134 × 210 × 4000	64.0	12.0	90.0	68	1461	349	0.24	38.1	300.0
	B1-S7(F)			Beam	Gradual	Yes	135 × 210 × 4000	64.0	12.0	90.0	68	1461	698	0.48	33.7	450.0
	B1-S7(J)			Beam	Gradual	Yes	136 × 210 ×4000	64.0	12.0	90.0	68	1461	698	0.48	34.0	450.0
	B2-S1(J)			Beam	Gradual	Yes	137 × 210 × 4000	64.0	12.0	90.0	68	1461	349	0.24	38.9	400.0
	B2-S1(F)			Beam	Gradual	Yes	138 × 210 × 4000	64.0	12.0	90.0	68	1461	349	0.24	39.0	400.0
	C1-S4(F)			Beam	Gradual	Yes	139 × 210 × 4000	62.0	16.0	180.0	63	1392	698	0.50	34.3	550.0
	C1-S4(J)			Beam	Gradual	Yes	140 × 210 × 4000	62.0	16.0	180.0	63	1392	698	0.50	34.6	550.0
	C1-S6/1			Beam	Gradual	Yes	141 × 210 × 4000	62.0	16.0	180.0	63	1392	698	0.50	31.3	600.0
	C1-S6/2			Beam	Gradual	Yes	142 × 210 × 4000	62.0	16.0	180.0	63	1392	698	0.50	31.9	600.0
	C1-S7/1			Beam	Gradual	Yes	143 × 210 × 4000	62.0	16.0	180.0	63	1392	698	0.50	31.6	550.0
	C1-S7/2			Beam	Gradual	Yes	144 × 210 × 4000	62.0	16.0	180.0	63	1392	698	0.50	32.2	550.0
	D1-1(J)		Sanded	Beam	Gradual	Yes	145 × 210 × 4000	63.3	13.5	90.0	68	1461	698	0.48	39.0	250.0
	D1-1(F)		Sanded	Beam	Gradual	Yes	146 × 210 ×4000	63.3	13.5	90.0	68	1461	698	0.48	39.1	250.0
	D1-S3(J)		Sanded	Beam	Gradual	Yes	147 × 210 × 4000	63.3	13.5	90.0	68	1461	698	0.48	39.2	200.0
	D1-S3(F)		Sanded	Beam	Gradual	Yes	148 × 210 × 4000	63.3	13.5	90.0	68	1461	698	0.48	39.4	200.0
[33]	A40-80-S	AFRP Arapee Tendon	Sanded	Beam	Gradual	Yes	160 × 300 × 6000	61.1	7.8	47.8	54	1134	419	0.37	27.0	220.0
	A40-80-L			Beam	Gradual	Yes	161 × 300 × 6000	61.1	7.8	47.8	54	1134	419	0.37	27.0	220.0
	A80-80-S			Beam	Gradual	Yes	164 × 300 × 6000	61.1	7.8	47.8	54	1134	419	0.37	64.0	170.0
	A80-80-L			Beam	Gradual	Yes	165 × 300 × 6000	61.1	7.8	47.8	54	1134	419	0.37	64.0	170.0
[35]	7.5S-N-50-1	AFRP Arapee Tendon	Sanded	Prism	Gradual	No	50 × 50 × 1000	21.3	7.5	22.2	91	3000	1487	0.50	54.6	-
	7.5S-N-60-1			Prism	Gradual	No	60 × 60 × 1000	26.3	7.5	22.2	91	3000	1487	0.50	54.6	135.0
	7.5S-N-70-1			Prism	Gradual	No	70 × 70 × 1000	31.3	7.5	22.2	91	3000	1487	0.50	54.6	135.0
	7.5S-N-50-2			Prism	Gradual	No	50 × 50 × 1000	21.3	7.5	22.2	91	3000	1487	0.50	54.6	220.0
	7.5S-N-60-2			Prism	Gradual	No	60 × 60 × 1000	26.3	7.5	22.2	91	3000	1487	0.50	54.6	110.0
	7.5S-N-70-2			Prism	Gradual	No	70 × 70 × 1000	31.3	7.5	22.2	91	3000	1487	0.50	54.6	135.0
	7.5S-N-50-3			Prism	Gradual	Fibres	50 × 50 × 1000	21.3	7.5	22.2	91	3000	1487	0.50	54.6	135.0
	7.5-S-N-50-4			Prism	Gradual	Fibres	50 × 50 × 1000	21.3	7.5	22.2	91	3000	1487	0.50	54.6	160.0
	7.5S-N-50-5			Prism	Gradual	Fibres	50 × 50 × 1000	21.3	7.5	22.2	91	3000	1487	0.50	54.6	160.0
	7.5S-H-45-1			Prism	Gradual	No	45 × 45 × 1000	18.8	7.5	22.2	91	3000	1487	0.50	81.5	110.0
	7.5S-H-50-1			Prism	Gradual	No	50 × 50 × 1000	21.3	7.5	22.2	91	3000	1487	0.50	81.5	135.0
	7.5S-H-50-2			Prism	Gradual	No	50 × 50 × 1000	21.3	7.5	22.2	91	3000	1487	0.50	81.5	135.0
	5.3S-N-45-1			Prism	Gradual	No	45 × 45 × 1000	19.9	5.3	11.1	91	3000	1487	0.50	54.6	85.0
	5.3E-N-45-1		Expancel	Prism	Gradual	No	45 × 45 × 1000	19.9	5.3	11.1	91	3000	1487	0.50	54.6	225.0
	5.3ES-N-45-1		Sanded	Prism	Gradual	No	45 × 45 × 1000	19.9	5.3	11.1	91	3000	1487	0.50	54.6	85.0
	5.3S-N-40-1		Sanded	Prism	Gradual	No	40 × 40 × 1000	17.4	5.3	11.1	91	3000	1487	0.50	54.6	85.0
	5.3E-N-40-1		Expancel	Prism	Gradual	No	40 × 40 × 1000	17.4	5.3	11.1	91	3000	1487	0.50	54.6	200.0
	5.3S-N-35-1		Sanded	Prism	Gradual	No	35 × 35 × 1000	14.9	5.3	11.1	91	3000	1487	0.50	54.6	85.0
	5.3S-N-30-1		Sanded	Prism	Gradual	No	30 × 30 × 1000	12.4	5.3	11.1	91	3000	1487	0.50	54.6	-
	5.3E-N-35-1		Expancel	Prism	Gradual	No	35 × 35 × 1000	14.9	5.3	11.1	91	3000	1487	0.50	54.6	185.0
	5.3E-N-30-1		Expancel	Prism	Gradual	No	30 × 30 × 1000	12.4	5.3	11.1	91	3000	1487	0.50	54.6	-
	5.3ES-N-35-1		Sanded	Prism	Gradual	No	35 × 35 × 1000	14.9	5.3	11.1	91	3000	1487	0.50	54.6	110.0
	5.3ES-N-30-1			Prism	Gradual	No	30 × 30 × 1000	12.4	5.3	11.1	91	3000	1487	0.50	54.6	-
	5.3S-N-40-2			Prism	Gradual	No	40 × 40 × 1000	17.4	5.3	11.1	91	3000	1487	0.50	54.6	-
	5.3S-N-35-2			Prism	Gradual	No	35 × 35 × 1000	14.9	5.3	11.1	91	3000	1487	0.50	54.6	85.0
	5.3-ES-N-40-1			Prism	Gradual	No	40 × 40 × 1000	17.4	5.3	11.1	91	3000	1487	0.50	54.6	135.0
	5.3ES-N-40-2			Prism	Gradual	No	40 × 40 × 1000	17.4	5.3	11.1	91	3000	1487	0.50	54.6	-
	5.3ES-N-35-2			Prism	Gradual	No	35 × 35 × 1000	14.9	5.3	11.1	91	3000	1487	0.50	54.6	110.0
	5.3ES-N-30-2			Prism	Gradual	No	30 × 30 × 1000	12.4	5.3	11.1	91	3000	1487	0.50	54.6	-
[47]	AA-2	AFRP Arapee Tendon	Smooth	Prism	Gradual	No	102 × 102 × 3000	46.1	9.9	38.1	128	2448	1027	0.42	31.6	482.6
	AF-2	AFRP Fibra	Smooth-braided	Prism	Gradual	No	103 × 102 × 3000	45.8	10.4	83.2	48	1434	807	0.56	30.8	330.2
	AT-2	AFRP Technora tendon	Rough	Prism	Gradual	No	104 × 102 × 3000	47.3	7.4	43.2	69	1724	884	0.51	30.1	345.4
	AA-1 (J)	AFRP Arapee Tendon	Smooth	Beam	Gradual	No	127 × 203 × 6000	63.7	9.9	38.1	128	2448	1040	0.43	31.5	406.4
	AA-1 (D)			Beam	Gradual	No	128 × 203 × 6000	63.7	9.9	38.1	128	2448	1040	0.43	31.5	266.7
	AA-3 (J)			Beam	Gradual	No	129 × 203 × 6000	63.7	9.9	38.1	128	2448	1437	0.59	29.0	685.8
	AA-3 (D)			Beam	Gradual	No	130 × 203 × 6000	63.7	9.9	38.1	128	2448	1437	0.59	29.0	711.2
	AF-3 (J)	AFRP Fibra	Smooth-braided	Beam	Gradual	No	131 × 203 × 6000	63.4	10.4	83.2	48	1434	828	0.58	29.7	381.0
	AF-3 (D)	AFRP Fibra	Smooth-braided	Beam	Gradual	No	132 × 203 × 6000	63.4	10.4	83.2	48	1434	828	0.58	29.7	304.8
	AT-1 (J)	AFRP Technora tendon	Rough	Beam	Gradual	No	133 × 203 × 6000	64.9	7.4	43.2	69	1724	1409	0.82	30.1	330.2
	AT-1 (D)			Beam	Gradual	No	134 × 203 × 6000	64.9	7.4	43.2	69	1724	1409	0.82	30.1	279.4
	AT-3 (J)			Beam	Gradual	No	135 × 203 × 6000	64.9	7.4	43.2	69	1724	976	0.57	30.1	254.0
	AT-3 (D)			Beam	Gradual	No	136 × 203 × 6000	64.9	7.4	43.2	69	1724	976	0.57	30.1	381.0
[75]	B1	AFRP Technora tendon	Rough	Beam	Sudden	Yes	150 × 300 × 2100	37.0	6.0	28.8	53	1767	1290	0.73	31.2	-
	B2			Beam	Sudden	Yes	151 × 300 × 2100	37.0	6.0	28.8	53	1767	1205	0.68	32.3	-
	B3			Beam	Sudden	Yes	152 × 300 × 2100	37.0	6.0	28.8	53	1767	1208	0.68	36.5	225.0
[53,54]	AT	AFRP Technora tendon	Rough	Beam	Gradual	No	89 × 178 × 3000	40.6	7.9	50.3	45	1710	1055	0.62	43.1	368.3

**Table A5:** Geometrical, mechanical properties of the material and transfer lengths of specimens with pretensioned BFRP reinforcement.

Reference	Specimen No	FRP Type	FRP Surface	Specimen Type	Release Type	Shear Reinforcement	Specimen Dimensions (b × h × l, mm)	c, mm	Ø, mm	A_p_, mm^2^	E_p_, GPa	f_pu_, MPa	f_pi_, MPa	f_pi_/f_pu_	f_ci_, MPa	L_t_, mm
[56]	B1S	BFRP Bar	Sanded	Beam	Sudden	No	100 × 200 × 3000	46.0	8.0	50.2	44	1126	381	0.34	27.0	350.0
	B1N			Beam	Sudden	No	100 × 200 × 3000	46.0	8.0	50.2	44	1126	365	0.32	27.0	500.0
	B2S			Beam	Sudden	No	100 × 200 × 3000	46.0	8.0	50.2	44	1126	346	0.31	27.0	250.0
	B2N			Beam	Sudden	No	100 × 200 × 3000	46.0	8.0	50.2	44	1126	350	0.31	27.0	400.0
[55]	Beam 1	BFRP Bar	Sanded	Beam	Gradual	Yes	90 × 200 × 3000	58.0	12.0	113.1	36	920	442	0.48	36.5	100.0
	Beam 2			Beam	Gradual	Yes	90 × 200 × 3000	58.0	12.0	113.1	36	920	442	0.48	52.4	93.0
